# A Descriptive Analysis of Oral Health Systematic Reviews Published 1991–2012: Cross Sectional Study

**DOI:** 10.1371/journal.pone.0074545

**Published:** 2013-09-30

**Authors:** Humam Saltaji, Greta G. Cummings, Susan Armijo-Olivo, Michael P. Major, Maryam Amin, Paul W. Major, Lisa Hartling, Carlos Flores-Mir

**Affiliations:** 1 Orthodontic Graduate Program, School of Dentistry, University of Alberta, Edmonton, Alberta, Canada; 2 Faculty of Nursing, University of Alberta, Edmonton, Alberta, Canada; 3 Faculty of Rehabilitation Medicine, University of Alberta, Edmonton, Alberta, Canada; 4 Division of Pediatric Dentistry, School of Dentistry, University of Alberta, Edmonton, Alberta, Canada; 5 Alberta Research Centre for Health Evidence, Department of Pediatrics, University of Alberta, Edmonton, Alberta, Canada; 6 Cochrane Child Health Field, Department of Pediatrics, University of Alberta, Edmonton, Alberta, Canada; Katholieke Universiteit Leuven, Belgium

## Abstract

**Objectives:**

To identify all systematic reviews (SRs) published in the domain of oral health research and describe them in terms of their epidemiological and descriptive characteristics.

**Design:**

Cross sectional, descriptive study.

**Methods:**

An electronic search of seven databases was performed from inception through May 2012; bibliographies of relevant publications were also reviewed. Studies were considered for inclusion if they were oral health SRs defined as therapeutic or non-therapeutic investigations that studied a topic or an intervention related to dental, oral or craniofacial diseases/disorders. Data were extracted from all the SRs based on a number of epidemiological and descriptive characteristics. Data were analysed descriptively for all the SRs, within each of the nine dental specialities, and for Cochrane and non-Cochrane SRs separately.

**Results:**

1,188 oral health (126 Cochrane and 1062 non-Cochrane) SRs published from 1991 through May 2012 were identified, encompassing the nine dental specialties. Over half (n = 676; 56.9%) of the SRs were published in specialty oral health journals, with almost all (n = 1,178; 99.2%) of the SRs published in English and almost none of the non-Cochrane SRs (n = 11; 0.9%) consisting of updates of previously published SRs. 75.3% of the SRs were categorized as therapeutic, with 64.5% examining non-drug interventions, while approximately half (n = 150/294; 51%) of the non-therapeutic SRs were classified as epidemiological SRs. The SRs included a median of 15 studies, with a meta-analysis conducted in 43.6%, in which a median of 9 studies/1 randomized trial were included in the largest meta-analysis conducted. Funding was received for 25.1% of the SRs, including nearly three-quarters (n = 96; 76.2%) of the Cochrane SRs.

**Conclusion:**

Epidemiological and descriptive characteristics of the 1,188 oral health SRs varied across the nine dental specialties and by SR category (Cochrane vs. non-Cochrane). There is a clear need for more updates of SRs in all the dental specialties.

## Introduction

A systematic review (SR) is a useful tool that serves to identify, appraise and integrate the findings of studies on a specific topic using a systematic approach. [1]–[3] It has become the gold standard for decision-making by clinicians and policy makers, and foundational to evidence-based practice approach. [2] Since the inception of the evidence-based practice approach in dentistry, the number of published SRs conducted in dental fields has rapidly increased. [4] One of the valuable sources for SRs is The Cochrane Collaboration, an international organization that aims to help health care professionals make well-informed decisions about treatment interventions by conducting high quality SRs. It has been acknowledged that SRs produced by this collaboration differ in their characteristics and reporting qualities from non-Cochrane SRs. [5]–[7].

In the field of oral health, there has been no comprehensive evaluation of all the published SRs. A few evaluations [8], [9] in the last decade have set out to examine characteristics of a sample of dental SRs; however the value of these evaluations is limited. Their limitations include: not examining all the pertinent epidemiologic and descriptive characteristics of oral health SRs; not considering the SR category (Cochrane *vs*. non-Cochrane) or the dental specialty in the analysis; not examining controversial areas relevant to SRs (e.g., publishing updates of SRs); nor providing a comprehensive evaluation of all the SRs published in the field of oral health research, but rather including a limited number of years in their searches (e.g., 2000 to 2005) and limiting it to the English language) [8].

Given the need for more evidence to guide informed decision-making by dental practitioners, the knowledge gained from a comprehensive description of all the oral health SRs and within each specialty would be of paramount importance. This work would help to: identify gaps where evidence is limited, as well as where more oral health SRs and further development are needed, direct future developments in the field of evidence-based dentistry, and provide information for future methodological and meta-epidemiological studies that are clearly needed to quantify the bias associated with methodologies in oral health randomized clinical trials. The purpose of this cross-sectional descriptive study is to provide a first step in the development of a database of all SRs published in the domain of oral health research. The objectives were to: (1) identify all of the oral health SRs published from inception through May 2012; and (2) describe the oral health SRs in terms of their epidemiological and descriptive characteristics.

## Materials and Methods

### Data Sources and Searches

Electronic searches up to May 2^nd^, 2012, were conducted using the following electronic bibliographic databases:

- PubMed (1966 to May 2012, week 1)
- MEDLINE (1980 to 2012, week 18)
- EMBASE (1980 to 2012, week 18)
- ISI Web of Science (1965 to May 2, 2012)
- Evidence-Based Medicine Reviews – Cochrane Database of Systematic Reviews (1991 to second quarter of 2012)
- Health STAR (1966 to May 2012).

The key words used in the search were “systematic review,” “meta-analysis,” “dentistry,” “tooth,” “orthodontics,” “oral surgery,” “endodontics,” “periodontics,” “prosthodontics,” “pedodontics,” “pediatric dentistry,” “dental public health,” and “oral pathology.” Subject subheadings and some word truncations, according to each database, were used as well to map all possible key words. The initial search strategy was designed for PubMed (Table 1) and adapted to other databases. The details of the specific search terms and combinations used in each individual database are listed in Table S1 in Appendix S1. The electronic searches were developed with the assistance of a librarian specializing in health science databases.

**Table 1:** Search Strategy in PubMed.

#1 systematic review* OR meta-analys*
#2 dent* OR tooth OR teeth OR orthodon* OR oral surg* OR endodon* OR periodon* OR prosthodon* OR pedodon* OR pediatric* dentistry OR paediatric* dentistry OR dent* public health OR oral pathology
#3 #1 AND #2

We also searched the American Dental Association (ADA)-Evidence-based Dentistry website [10] on May 18–20, 2012. In addition, we have searched the bibliographies of articles that focused on the quality of SRs in the dental fields. The searches were not limited to the English language nor restricted by other means. The references resulting from the searches were entered in EndNote X5, and duplicates were removed.

### Study Selection and Data Extraction

Appropriate reports to be included met the following pre-established eligibility criteria:

- Reports fit within the following definition: Oral health SR was defined as one that studied a therapeutic or non-therapeutic topic related to dental, oral or craniofacial diseases/disorders as defined by the ADA scope of practice. [11] We considered a report to be a SR if the authors set out to summarize evidence from several studies and reported explicit methods to identify and evaluate relevant studies. [9], [12]
- The SR should be a full-length report.
- SRs in all languages were eligible.
- If a duplicate involving a Cochrane SR and a non-Cochrane SR generated from it was identified, only the Cochrane SR was included.

Two researchers (H.S & T.K) independently reviewed the list of titles and abstracts for inclusion. Once potentially relevant abstracts were selected, the full reports were retrieved for a final selection process. If the abstract was judged to contain insufficient information to ascertain the appropriateness of the work for inclusion, the full report was obtained and reviewed before a final decision was made. Any discrepancies in the inclusion of reports between researchers were addressed through discussion until a consensus was reached. The selected SRs were classified according to one of the following dental specialties as defined by the ADA [11]:

- Dental public health
- Endodontics
- Oral medicine and pathology
- Oral and maxillofacial radiology
- Oral and maxillofacial surgery
- Orthodontics and dentofacial orthopedics
- Pediatric dentistry
- Periodontics
- Restorative dentistry and prosthodontics.

We modified the ADA classification [11] by adding oral medicine to “oral and maxillofacial pathology”, and “restorative dentistry” to “prosthodontics”.

A data extraction template was designed using Microsoft Excel and pilot tested. Data were extracted on the following characteristics: [5], [8], [13] dental specialty, year of publication, country of corresponding author, continent of corresponding author, number of authors, number of schools/affiliations, career type of the primary author (e.g., academic, private practice, public health, industry), name of journal, type of journal (e.g., general dentistry, specialty dentistry, non-dental), impact factor of journal, source of funding (e.g., industry, government, foundation, academic), type and focus of review (e.g., therapeutic, non- therapeutic: diagnosis/prognosis, epidemiology, psychological/educational), nature of intervention (e.g., drug, surgical, device, dental material, psychological, educational, policy), language of review, design of included studies, number of included studies, number of included randomized controlled trials (RCTs), whether eligible studies were found, whether a meta-analysis (MA) was conducted, number of studies and RCTs contributing data to the largest MA conducted, and whether the review is an update of a previous report.

Complete data extraction was achieved by a non-blinded assessor (H.S), among which a random sample of roughly 20% (250 SRs) was performed in duplicate by two assessors (H.S & T.K/M.A) to assess accuracy. Discrepancies were resolved through discussion until a consensus was reached.

### Data Analysis

Data were analyzed descriptively as frequency, median, or interquartile range (IQR). The data were analysed for all the SRs, within each of the dental specialities, and for Cochrane and non-Cochrane SRs separately. Data analysis was performed using the Statistical Package for Social Sciences (SPSS, Version 18.0; IBM, Armonk, NY) for Windows (Microsoft Corporation, Redmond, WA).

## Results

### Literature Search

**Figure 1:**
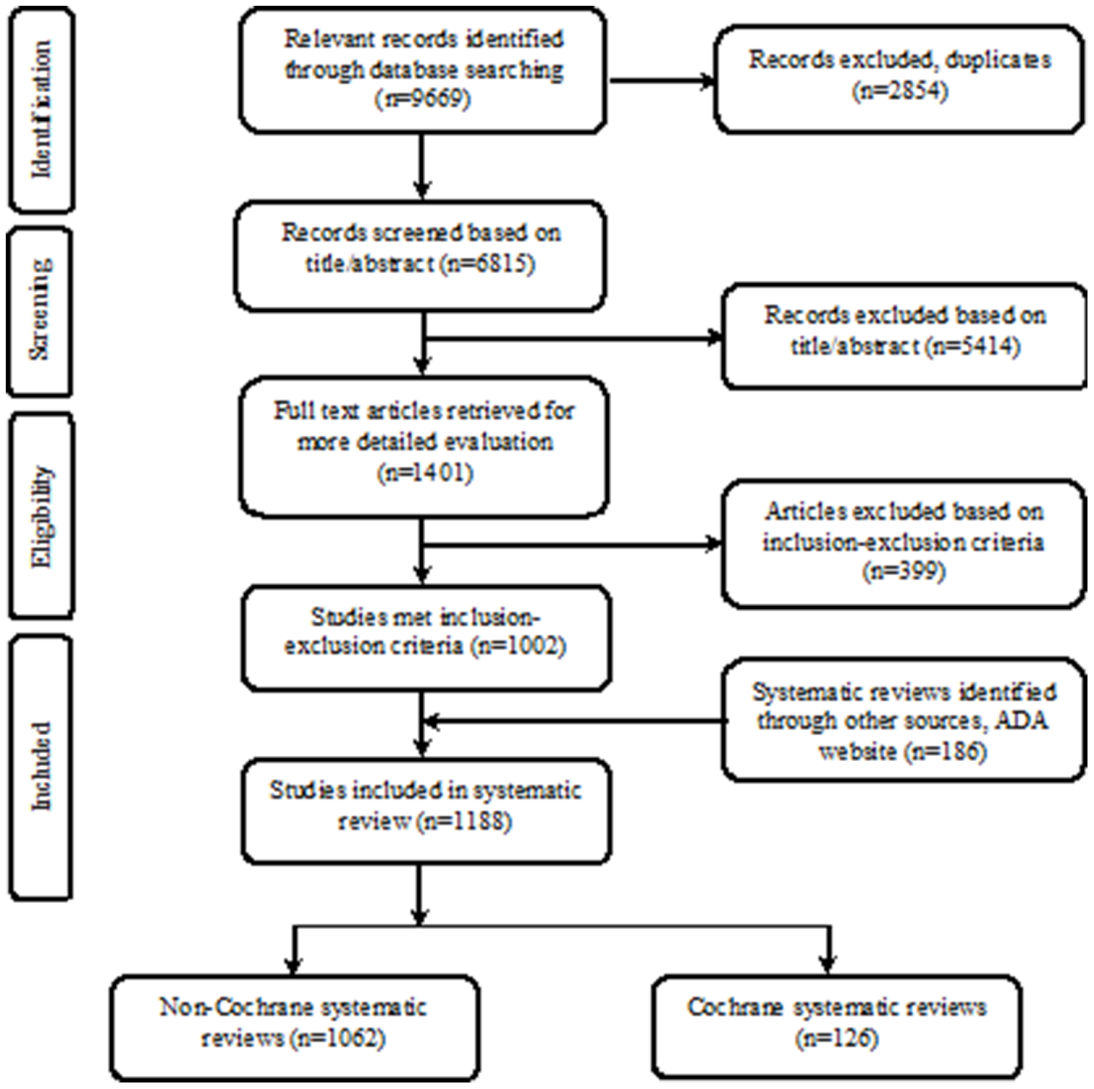
Flow Diagram of the Literature Search According to the PRISMA [**28**].

The search returned 9669 potential records for inclusion, including 2854 duplicates. The search results from different electronic databases are listed in Table S1 in Appendix S1. Through the process of screening, 5414 records were excluded based on title/abstract. The remaining 1401 full-text reports were retrieved for a more detailed evaluation, of which 1002 reports fulfilled the inclusion-exclusion criteria. An additional 186 reports were identified through the ADA-Evidence-based Dentistry website [10] search or reference list search, and 1188 reports were finally included. A flow diagram of the data search is given in Figure 1. The main reasons for exclusion were not being within the scope of any of the dental fields or not using explicit methods to identify relevant studies.

### Prevalence and Specialties of Oral Health SRs

The majority of the SRs were published either in the fields of periodontics (n = 212; 17.8%), prosthodontics and restorative dentistry (n = 198; 16.7%), or dental public health (n = 184 = 15.5%). Oral health SRs published in the remaining dental specialities included: oral medicine and oral pathology (n = 162; 13.6%), oral and maxillofacial surgery (n = 59; 13.4%), orthodontics and dentofacial orthopedics (n = 138; 11.6%), endodontics (n = 54; 4.5%), pediatric dentistry (n = 50; 4.2%), and oral and maxillofacial radiology (n = 31; 2.6%). Table 2 provides further details of the number of oral health SRs within each of the nine dental specialities and for Cochrane and non-Cochrane SRs separately.

**Table 2:** Specialties of Oral Health Systematic Reviews, N (% Total).

Dental Specialty	Overall (Cochrane & Non-Cochrane SRs) N = 1188	Non-Cochrane SRs (N = 1062)	Cochrane SRs (N = 126)
Periodontics	212 (17.8)	203 (19.1)	9 (7.1)
Prosthodontics & Restorative Dentistry	198 (16.7)	179 (16.9)	19 (15.1)
Dental Public Health	184 (15.5)	163 (15.3)	21 (16.7)
Oral Medicine & Oral Pathology	162 (13.6)	140 (13.2)	22 (17.5)
Oral and Maxillofacial Surgery	159 (13.4)	134 (12.6)	25 (19.8)
Orthodontics and Dentofacial Orthopedics	138 (11.6)	123 (11.6)	15 (11.9)
Endodontics	54 (4.5)	47 (4.4)	7 (5.6)
Pediatric Dentistry	50 (4.2)	42 (4.0)	8 (6.3)
Oral and Maxillofacial Radiology	31 (2.6)	31 (2.9)	0 (0.0)
**Total**	1188 (100)	1062 (100)	126 (100)

### Characteristics of Oral Health SRs

**Figure 2:**
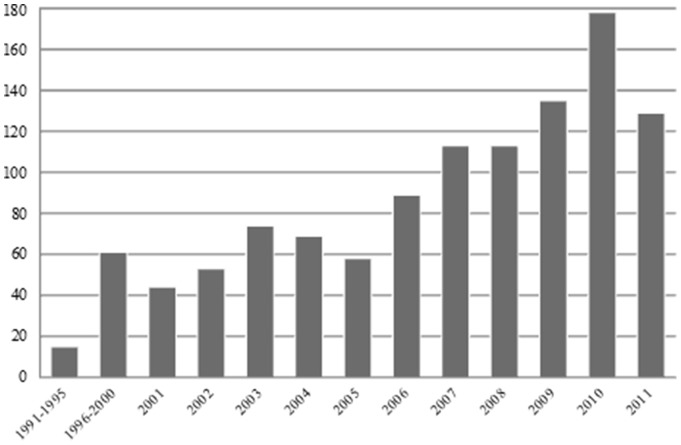
Number of Systematic Reviews Published by Year; 2012 Was Not Included in the Figure because the Full Year Was Not Searched (Y Axis Represents Numbers of Reviews).

The 1188 SRs were published between 1991 and 2012. The median date of publication of oral health SRs was 2008, ranging from 2006 for dental public health publications to 2009 for oral and maxillofacial radiology publications. Figure 2 shows the increase of oral health SRs, with each year, from 1991 to 2011. The majority of the published SRs were non-Cochrane SRs (n = 1062; 89.4%), while Cochrane SRs contributed only 10% of the total number of SRs (n = 126; 10.6%).

The SRs were published in 194 (96 oral health & 98 non-oral health) journals. More than half of the SRs were published in specialty oral health journals (n = 676; 56.9%), while 373 SRs (31.4%), including all of the Cochrane SRs, were published in general oral health journals. Nearly one third of the non-Cochrane SRs (n = 335; 32%) were published in eight (one general and seven specialty) oral health journals, namely the *Journal of Clinical Periodontology* (n = 75; 6.3%), *Clinical Oral Implants Research* (n = 59; 5.0%), *the Journal of Periodontology* (n = 40; 3.4%), the *Angle Orthodontist* (n = 35; 2.9%), the *American Journal of Orthodontics and Dentofacial Orthopedics* (n = 34; 2.9%), *the International Journal of Oral & Maxillofacial implants* (n = 34; 2.9%), the *Journal of Oral and Maxillofacial Surgery* (n = 30; 2.5%), and *the Journal of the American Dental Association* (n = 28; 2.4%) (Table 3). Almost half of the non-Cochrane SRs (n = 489; 47%) were published in journals with a relatively high impact factor for the field of dentistry (>1.5); while 7.1% (n = 84) of the non-Cochrane SRs were published in oral health journals that did not have an impact factor (Table 4).

**Table 3:** Journals in which Oral Health Systematic Reviews Were Published.

Journal Title	Classification	No. (%) of 1188 SRs (Cochrane and Non-Cochrane SRs)	Rank†	Impact Factor†
*1. Journal of Clinical Periodontology*	Specialty	75 (6.3)	5	2.996
*2. Clinical Oral Implants Research*	Specialty	59 (5.0)	13	2.514
*3. Journal of Periodontology*	Specialty	40 (3.4)	11	2.602
*4. Angle Orthodontist*	Specialty	35 (2.9)	40	1.207
*5. American Journal of Orthodontics and Dentofacial Orthopedics*	Specialty	34 (2.9)	35	1.381
*5. The International Journal of Oral & Maxillofacial implants*	Specialty	34 (2.9)	21	1.776
*6. Journal of Oral and Maxillofacial Surgery*	Specialty	30 (2.5)	27	1.640
*7. The Journal of the American Dental Association*	General	28 (2.4)	22	1.773
*8. Journal of Endodontics*	Specialty	26 (2.2)	7	2.880
*8. Journal of Dentistry*	General	26 (2.2)	6	2.947
*9. Journal of Dental Research* ‡	General	25 (2.1)	3	3.486
*9. Oral Surgery Oral Medicine Oral Pathology Oral Radiology and Endodontology*	General	25 (2.1)	33	1.457
*10. The International journal of prosthodontics*	Specialty	24 (2.0)	36	1.376
*11. Journal of Dental Education*	Specialty	23 (1.9)	61	0.906
*12. The Journal of Prosthetic Dentistry*	Specialty	21 (1.8)	37	1.324
*13. Journal of Oral Rehabilitation*	Specialty	19 (1.6)	30	1.529
*14. Community Dentistry and Oral Epidemiology*	Specialty	18 (1.5)	19	1.894
*15. Dentomaxillofacial Radiology*	Specialty	16 (1.3)	49	1.081
*16. International journal of oral and maxillofacial surgery*	Specialty	14 (1.2)	32	1.506
*17. International Journal of Dental Hygiene*	Specialty	13 (1.1)	63	0.871
*18. Dental Materials*	Specialty	12 (1.0)	4	3.135
*18. International Dental Journal*	General	12 (1.0)	58	0.963
*18. Acta odontologica scandinavica*	General	12 (1.0)	50	1.066
*Cochrane Database of Systematic Reviews*	General	126 (10.6)	N/A	5.912
Other oral health journals (with IF)	General/Specialty	218 (18.3)	-	-
Other oral health journals (IF is not found)	General/Specialty	84 (7.1)	-	Not found
Non-oral health journals	Non-dental	139 (11.7)	-	-
Total number of oral health journals (1094 SRs)	96 (63 with IF & 33 without IF)			
Total number of non-oral health journals (139 SRs)	98			

† 2011 Journal Citation Reports® (Thomson Reuters, 2012).

‡ SRs published in *Critical Reviews in Oral Biology & Medicine* were included in *Journal of Dental Research*. *Critical Reviews in Oral Biology & Medicine* was merged into the *Journal of Dental Research* (last issue Nov 2004).

IF, impact factor; N/A, not applicable.

**Table 4:** Characteristics of Oral Health Systematic Reviews.

Characteristic	No. (%) of 1188 SRs (Cochrane and Non-Cochrane SRs)	No. (%) of 126 Cochrane SRs	No. (%) of 1062 Non-Cochrane SRs
**Year of publication, median**			
	2008	Protocol: 2004; Review: 2007	2008
**Continent of corresponding author, n (% total)**			
*Europe*	645 (54.3)	99 (78.6)	546 (51.4)
*North America*	303 (25.5)	2 (1.6)	301 (28.3)
*Asia*	99 (8.3)	13 (10.3)	86 (8.1)
*South America*	61 (5.1)	10 (7.9)	51 (4.8)
*Australia*	47 (4.0)	0 (0.0)	47 (4.4)
*Africa*	33 (2.8)	2 (1.6)	31 (2.9)
**Country of corresponding author, n (% total)**			
*No. of countries*	47	20	47
*USA*	218 (18.4)	1 (0.8)	217 (20.4)
*UK*	196 (16.5)	82 (65.1)	114 (10.7)
*Canada*	85 (7.2)	1 (0.8)	84 (7.9)
*The Netherlands*	82 (6.9)	1 (0.8)	81 (7.6)
*Switzerland*	67 (5.6)	0 (0.0)	67 (6.3)
*Italy*	65 (5.5)	4 (3.2)	61 (5.7)
*Brazil*	57 (4.8)	9 (7.1)	48 (4.5)
*Germany*	46 (3.9)	4 (3.2)	42 (4.0)
*Sweden*	40 (3.4)	0 (0.0)	40 (3.8)
*China*	40 (3.4)	5 (4.0)	35 (32.9)
*Greece*	28 (2.4)	0 (0.0)	28 (2.6)
*Australia*	28 (2.4)	0 (0.0)	28 (2.6)
*Spain*	25 (2.1)	0 (0.0)	25 (2.4)
*South Africa*	25 (2.1)	1 (0.8)	24 (2.3)
*Other*	186 (15.6)	18 (14.3)	168 (15.8)
**Career type of the primary author, n (% total)**			
Academic	1084 (91.2)	105 (83.3)	979 (92.2)
Private practice	47 (4.0)	5 (4)	42 (4)
Policy/Public health	39 (3.3)	16 (2.7)	23 (2.2)
Industry	18 (1.5)	0 (0.0)	18 (1.7)
**Journal impact factor** ‡ **, n (% total)**			
0.0–1.000	122 (10.3)	0 (0.0)	122 (11.5)
1.001–1.500	219 (18.4)	0 (0.0)	219 (20.6)
1.501–2.000	170 (14.3)	0 (0.0)	170 (16.0)
2.001–3.000	282 (23.7)	0 (0.0)	282 (26.5)
3.001–4.000	46 (3.9)	0 (0.0)	46 (4.3)
4.001§<	126 (10.6)	126 (100)	0 (0.0)
Not found*	84 (7.1)	0 (0.0)	84 (7.9)
N/A¶	139(11.7)	0 (0.0)	139 (13.1)
**Journal type** † **, n (% total)**			
General Dentistry	373 (31.4)	126 (100.0)	247 (23.3)
Specialty Dentistry	676 (56.9)	0 (0.0)	676 (63.7)
Non-Dental	139 (11.7)	0 (0.0)	139 (13.1)
**Language, n (% total)**			
English	1178 (99.2)	126 (100.0)	1052 (99.1)
Bilingual English	6 (0.5)	0 (0.0)	6 (0.6)
Other	4 (0.3)	0 (0.0)	4 (0.4)
**Update of previous review** ‡ **, n (% total)**			
Yes	11 (0.9)	N/A	11 (1.0)
No	1051 (88.5)	N/A	1051 (99.0)
**Number of databases, n (% total)**			
1–2	518 (43.6)	1 (0.8)	517 (48.7)
3–4	373 (31.4)	62 (49.2)	311 (29.3)
>4	253 (21.3)	63 (50.0)	190 (17.9)
Unclear/Not reported	44 (3.7)	0 (0.0)	44 (4.1)

‡ 2011 Journal Citation Reports® (Thomson Reuters, 2012). The highest impact factor for oral health journals is 3.961 (*Periodontology 2000*).

† Cochrane Database of Systematic Reviews (CDSR), where Cochrane SRs are published, was classified as a general journal.

§ Includes Cochrane SRs only (CDSR’s impact factor = 5.912).

* Includes SRs published in oral health journals without impact factor.

¶ Includes SRs published in non-oral health journals.

‡ Does not equal 100% for overall, as Cochrane SRs were not considered in the analysis.

N/A, not applicable.

The corresponding authors of the SRs were most frequently from Europe (Cochrane SRs: n = 99; 78.6% & non-Cochrane SRs: 546; 51.4%) followed by North America, with one country (UK) accounting for nearly two-thirds (n = 82; 65.1%) of the Cochrane SRs, another country (USA) accounting for nearly one-quarter (n = 217; 20.4%) of the non-Cochrane SRs, and four countries (the United States, the United Kingdom, Canada, and the Netherlands) accounting for nearly half (n = 581; 48.9%) of all oral health SRs (Table 4). Approximately half of the SRs had authors from multiple centers (median of two affiliations for non-Cochrane SRs and three affiliations for Cochrane SRs), and included four to six authors (median of three authors for non-Cochrane SRs and five authors for Cochrane SRs) although 78 (7.3%) of the non-Cochrane SRs were single-authored (Table 5 & Table S4 in Appendix S1). The primary authors were from an academic background in the vast majority of the oral health SRs (n = 1084; 91.2%), with a small proportion published by private practice clinicians (n = 47; 4.0%), researchers from policy/public health organizations (n = 39; 3.3%), and researchers from dental companies (n = 18; 1.5%).

**Table 5:** Characteristics of Oral Health Systematic Reviews.

Characteristic	No. (%) of 1188 SRs (Cochrane and Non- Cochrane SRs)	No. (%) of 126 Cochrane SRs	No. (%) of 1062 Non- Cochrane SRs
**Number of Authors**			
Number of authors, median (IQR)			
	4 (2, 5)	5 (4, 6)	3 (2, 5)
Number of authors, n (% total)			
1	78 (6.6)	0 (0.0)	78 (7.3)
2–3	505 (42.5)	26 (20.6)	479 (45.1)
4–6	520 (43.8)	81 (64.3)	439 (41.3)
≥ 7	85 (7.2)	19 (15.1)	66 (6.2)
**Number of Schools/Affiliations**			
Number of schools, median (IQR)			
	2 (1, 3)	3 (2, 4)	2 (1, 3)
Number of schools, n (% total)			
1	454 (38.2)	19 (15.1)	435 (41.0)
2–3	573 (48.2)	57 (45.2)	516 (48.6)
4≤	161 (13.6)	50 (39.7)	111 (10.5)
**Type of Review, N (% Total)**			
Therapeutic	894 (75.3)	126 (100)	768 (72.3)
Non-therapeutic	294 (24.7)	0 (0.0)	294 (27.7)
**Focus of Non-therapeutic SRs, N (% Total)**			
*Total Number*	N = 294	N = 0	N = 294
Diagnosis/Prognosis	112 (38.1)	0 (0.0)	112 (38.1)
Epidemiology	150 (51)	0 (0.0)	150 (51)
Psychological/Educational/Policy/Quality of studies	32 (10.9)	0 (0.0)	32 (10.9)
**Type of Intervention in Therapeutic SRs, N (% Total)**			
Classification I, N (% Total)			
*Total Number*	N = 894	N = 126	N = 768
Drug	219 (24.5)	34 (27.0)	185 (24.1)
Non-drug	577 (64.5)	74 (58.7)	503 (65.5)
Both	98 (11.0)	18 (14.3)	80 (10.4)
Classification II, N (% Total)			
*Total Number*	N = 894	N = 126	N = 768
Surgical	151 (16.9)	25 (19.8)	126 (16.4)
Non-surgical	651 (72.8)	96 (76.2)	555 (72.3)
Both	92 (10.3)	5 (4.0)	87 (11.3)
Classification III, N (% Total)			
*Total Number*	N = 894	N = 126	N = 768
Surgical	145 (16.2)	22 (17.5)	123 (16.0)
Device	163 (18.2)	12 (9.5)	151 (19.7)
Drug	194 (21.7)	35 (27.8)	159 (20.7)
Dental Material	96 (10.7)	12 (9.5)	84 (10.9)
Psychological/Educational/Policy	31 (3.5)	7 (55.6)	24 (3.1)
Other	105 (11.7)	22 (17.5)	83 (10.8)
Multiple/Combined	160 (17.9)	16 (12.7)	144 (18.7)
**Source of Funding, N (% Total)**			
Classification I, N (% Total)			
Yes	298 (25.1)	96 (76.2)	202 (19.0)
No	58 (4.9)	1 (0.8)	57 (5.4)
Not reported	832 (70.0)	29 (23)	803 (75.6)
Classification II, N (% Total)			
*Total Number*	N/A	N = 48‡	N = 202
Industry	-	1 (2.1)	20 (9.9)
Government	-	7 (14.6)	37 (18.3)
Foundation	-	30 (62.5)	67 (33.2)
Academic	-	1 (2.1)	41 (20.3)
Multiple	-	9 (18.8)	33 (16.3)
Unclear	-	0 (0.0)	4 (2.0)
Classification III, N (% Total)			
Internal only	-	49 (38.9)	-
External only	-	6 (4.8)	-
Both internal and external	-	41 (32.5)	-
Not reported	-	29 (23.0)	-
No	-	1 (0.8)	-

‡ External funding only; N/A, not applicable.

Three-quarters (n = 894; 75.3%) of the SRs, including all the Cochrane SRs, were categorized as therapeutic; the vast majority (approximately 90%) of the SRs in the fields of prosthodontics and restorative dentistry, oral and maxillofacial surgery, and endodontics were categorized as therapeutic, and the vast majority (n = 29; 93.5%) of the SRs in the field of oral and maxillo-facial radiology were categorized as non-therapeutic. Approximately half (n = 150/294; 51%) of the non-therapeutic SRs were classified as epidemiology SRs, including the majority (n = 56/82; 68.3%) of the SRs in the field of oral medicine and oral pathology, and 38.1% (112/294) as diagnostic/prognostic SRs, including the vast majority (n = 25/29; 86.2%) of the SRs in the field of oral and maxillo-facial radiology (Table 5 & Table S4 in Appendix S1).

The nature of intervention varied across the dental specialties, with nearly two-thirds (n = 577/894; 64.5%) of all the therapeutic SRs examining non-drug interventions, including the vast majority (approximately 90%) of the therapeutic SRs in the fields of prosthodontics and restorative dentistry, and orthodontics and dentofacial orthopedics. Nearly three-quarters (n = 651/894; 72.8%) of all the therapeutic SRs examined non-surgical interventions, including almost all of the therapeutic SRs in the fields of dental public health and pediatric dentistry. Moreover, similar ratios of therapeutic SRs reported examining surgical (n = 145/894; 16.2%), device (n = 163/894; 18.2%), drug (n = 194/894; 21.7%), and multiple (n = 160/894; 17.9%) interventions, with a small portion (n = 31/894; 3.5%) examining psychological or educational interventions (Table 5 & Table S4 in Appendix S1).

**Figure 3:**
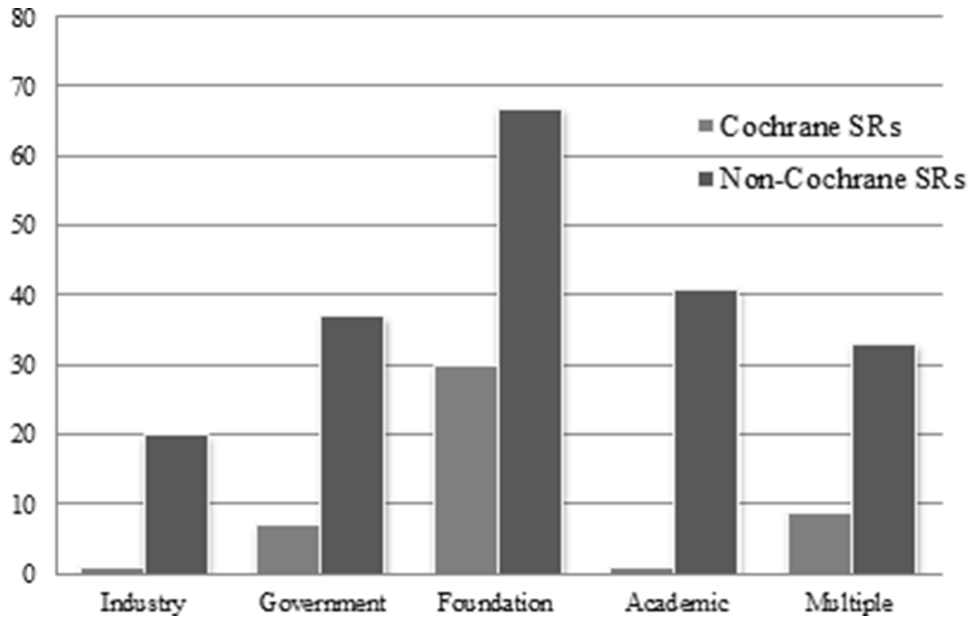
Number of Oral Health Systematic Reviews by Source of Funding.

One-quarter (n = 298; 25.1%) of all the SRs, including nearly three-quarters (n = 96; 76.2%) of the Cochrane SRs, reported receiving at least one source of funding. Approximately one-third (n = 66/184; 35.9%) of the SRs in the field of dental public health, including all (n = 21/21; 100%) the Cochrane SRs, received funding, while only a small portion (n = 2/31; 6.5%) of the SRs in the field of oral and maxillo-facial radiology reported receiving funding. The most common sources of funding for non-Cochrane SRs were foundations (n = 67/202; 33.2%) followed by academic (n = 41/202; 20.3%) and government (n = 37/202; 18.3%) sources. For Cochrane SRs, nearly three-quarters (n = 90; 71.4%) reported receiving an external source of funding, with “foundations” as the most common (30/48; 62.5%) external source of funding (Table 5 & Figure 3).

Almost all (n = 1178; 99.2%) of the SRs were published in English, and almost none of the non-Cochrane SRs (n = 11; 0.9%) were updates of previously published SRs (Table 4). While almost all the Cochrane SRs included RCTs only (n = 97/126; 93.3%), only 17.6% (n = 186/1062) of the non-Cochrane SRs included only RCTs. The research design of studies included in non-Cochrane SRs were most often non-RCTs (n = 423; 39.9%), including the majority of the SRs in the fields of oral and maxillo-facial radiology (n = 25/31; 80.6%) and oral medicine and oral pathology (83/140; 59.3%), followed by RCTs and other designs (n = 325; 30.7%) and RCTs only (n = 186; 17.6%).

Non-Cochrane SRs included a median of 15 studies, ranging from 12 for orthodontics and dentofacial orthopedics to 16.5 for oral medicine and oral pathology; while the median number of studies included in Cochrane SRs was five, ranging from two for oral medicine and oral pathology to twelve for dental public health (Table 6 & Table S6 in Appendix S1). The median number of RCTs included in the non-Cochrane SRs was one, ranging from zero for oral medicine and & oral pathology, pediatric dentistry and orthodontics and dentofacial orthopedics to four for dental public health, while the Cochrane SRs included a median of five RCTs, ranging from two for orthodontics and dentofacial orthopedics and oral and maxillofacial surgery to twelve for dental public health. There were no eligible studies in 22 (17.5%) of the Cochrane SRs, while only three (0.3%) of the non-Cochrane SRs included no relevant studies.

**Table 6:** Characteristics of Included Studies in Oral Health Systematic Review.

Characteristic	No. (%) of 1188 SRs (Cochrane and Non-Cochrane SRs)	No. (%) of 126 Cochrane SRs	No. (%) of 1062 Non-Cochrane SRs
**Study Designs of SRs with Eligible Studies, N (% Total)**			
*Total Number*	N = 1163	N = 104	N = 1059
RCTs only	283 (24.3)	97 (93.3)	186 (17.6)
CCTs only	10 (0.9)	1 (1.0)	9 (0.8)
RCTs and CCTs	71 (6.1)	4 (3.8)	67 (6.3)
RCTs and other designs	326 (28.0)	1 (1.0)	325 (30.7)
Non-RCTs	424 (36.5)	1 (1.0)	423 (39.9)
Unclear/Not reported	49 (4.2)	0 (0.0)	49 (4.6)
**Number of Included Studies**			
Number of included studies, median (IQR)			
	14 (7, 28)	5 (1, 13)	15 (8, 29)
Number of included studies, n (% total)			
0	25 (2.1)	22 (17.5)	3 (0.3)
1–5	166 (14.0)	45 (35.7)	121 (11.4)
6–15	433 (36.4)	32 (25.4)	401 (37.8)
16–30	261 (22.0)	17 (13.5)	244 (23.0)
>30	251 (21.1)	10 (7.9)	241 (22.7)
Unclear/Not reported	52 (4.4)	0 (0.0)	52 (4.9)
**Number of Included RCTs**			
Number of included RCTs, median (IQR)			
	1 (0, 7)	5 (1, 12)	1 (0, 6)
Number of included RCTs, n (% total)			
0	461 (38.3)	24 (19)	437 (41.1)
1–2	116 (9.8)	27 (21.4)	89 (8.4)
3–4	72 (6.1)	11 (8.7)	61 (5.7)
5–10	183 (15.4)	27 (21.4)	156 (14.7)
11–20	96 (8.1)	18 (14.3)	78 (7.3)
>20	75 (75)	19 (15.1)	56 (5.3)
Unclear/Not reported	185 (15.6)	0 (0.0)	185 (17.4)
**Meta-Analysis Conducted, N (% Total)**			
Yes	518 (43.6)	64 (50.8)	454 (42.7)
No	670 (56.4)	62 (49.2)	608 (57.3)
**Number of Studies Contributed Data to the Largest Meta-Analysis Conducted**			
*Total Number*	N = 518	N = 64	N = 454
Number of studies in largest meta-analysis, median (IQR)			
	9 (5, 18)	5.5 (3, 9)	9 (6, 19)
Number of studies in largest meta-analysis, n (% total)			
*Total Number*	N = 518	N = 64	N = 454
2–4	100 (19.3)	31 (48.4)	69 (15.2)
5–10	200 (38.6)	20 (31.2)	180 (39.6)
11–20	108 (20.8)	7 (10.9)	101 (22.2)
>20	104 (20.1)	6 (9.4)	98 (21.6)
Unclear/Not reported	6 (1.2)	0 (0.0)	6 (1.3)
Number of RCTs in largest meta-analysis, median (IQR)			
	2 (0, 6)	4.5 (2, 9)	1 (0, 6)
Number of RCTs in largest meta-analysis, n (% total)			
0	188 (36.3)	0 (0.0)	188 (41.4)
2–4	107 (20.7)	32 (50.0)	75 (16.5)
5–10	104 (20.1)	19 (29.7)	85 (18.7)
11–20	27 (5.2)	7 (10.9)	20 (4.4)
>20	21 (4.1)	6 (9.4)	15 (3.3)
Unclear/Not reported	71 (13.7)	0 (0.0)	71 (15.6)

RCTs, randomized controlled trials; CCTs, controlled clinical trials; N/A, not applicable.

Less than half of the SRs (n = 51; 43.6%) conducted quantitative analyses (meta-analyses). A median of nine studies and a median of two RCTs were included in the largest MA conducted (Table 6 & Table S7 in Appendix S1). This varied across dental specialties and the category of the review, with a median of 5.5 studies and 4.5 RCTs included in the largest MA conducted in the Cochrane SRs, and a median of nine studies and one RCT included in the largest MA conducted in the non-Cochrane SRs. 152 (29.4%) SRs (32 Cochrane and 120 non-Cochrane), in which a MA was conducted, included at least five RCTs. Tables S2 to S7 in Appendix S1 provide further details of the epidemiological and descriptive characteristics of all of the oral health SRs, within each of the dental specialities, and for Cochrane and non-Cochrane SRs separately.

## Discussion

SRs are important tools for researchers, clinicians and policy makers because they serve to systematically identify and appraise the available evidence on a specific topic, and to integrate it into an evidence-based conclusion. [1]–[3] This study demonstrates variation in the characteristics of SRs across the nine dental specialties and according to SR category (Cochrane *vs*. non-Cochrane). Our findings shows that the number of SRs published in the domain of oral health research and within each dental specialty has steadily increased over the last two decades, similar to the results published in previous reports examining dental SRs [9], [12], [14] and medical SRs [5], [13], [15]. However, there was a decline observed in 2011, which was also observed in previously published reports, [12], [13] and could be attributed to the fact that oral health SRs published in late 2011 would not necessarily be indexed by May 2^nd^, 2012, a so called time lag. The increased volume of SRs may not necessarily reflect a steady improvement in the methodological quality of the published SRs though. Previously published reports demonstrated that oral health SRs improved as a whole over a period of five years, [8], [12] with some specialities (e.g., periodontics) performing better at meeting the methodological quality criteria. [12] In order to avoid biased results and misleading decision-making in the dental practice, it is necessary that the increase in the quantity of published dental SRs be associated with an increase in the methodological quality of these SRs. Our study did not provide detailed information on methodological quality criteria, as our overall goal was to provide the reader with a detailed descriptive analysis of all SRs published in the field of dentistry.

Dental specialities were ranked according to the proportion of the total published SRs as follows (in descending order): periodontics, prosthodontics and restorative dentistry, dental public health, oral medicine and oral pathology, oral and maxillo-facial surgery, orthodontics and dentofacial orthopedics, endodontics, pediatric dentistry, and oral and maxillofacial radiology. Despite the steady increase in the number of published oral health SRs, there have only been a few SRs published in the fields of oral and maxillofacial radiology (31 SRs), pediatric dentistry (50 SRs), and endodontics (54 SRs); therefore, more SRs are specifically needed in these fields. However, it should be noted that many pediatric-related SRs were found to be better classified in the field of dental public health (e.g., “Fluoride supplements for preventing dental caries in children” [16]); ergo it is likely that the resulting number of published pediatric dental SRs in this study are underestimated and may not be representative of reality. Additionally, given that the ADA classification [11] was utilized for categorizing the selected SRs, implantology-related SRs were not classified in an individual field, but in one of three specialties (periodontics, oral and maxillofacial surgery, or prosthodontics). Given that the field of implantology is a relatively new and quickly growing dental field, future studies should consider it as an individual dental specialty in order not to inflate the SR count of other specialties.

Oral health SRs appear to be published more often in specialty journals. Our results showed that more than half of the SRs were published in specialty oral health journals, with almost half of the SRs published in journals with a high impact factor. Nearly half of the SRs were from four countries: the United States, the United Kingdom, Canada, and the Netherlands. This trend is similar to what was found in recently published reports, [14], [17] and could be attributed to an increased interest of the public sector and government agencies in these countries to make decisions regarding financing dental services based on the findings of the SRs. [14].

The current study revealed that many characteristics of the published oral health SRs still require improvement. For example, only 11 out of the 1062 non-Cochrane SRs were updates of previously published SRs. Furthermore, none of the 11 updates identified in our research were considered “up-to-date” according to the Cochrane policy, which requires updating the SR every two years. [6] This is a disappointing fact given that “up-to-date” evidenced-based conclusions are considered essential for decision making. [18] This might be explained by the fact that updates are usually given lower priority by funding agencies and editors, who tend not to publish updates with results that are the same as previously published versions. [5], [18] Therefore, updates of SRs in the domain of oral health research are clearly needed. In light of this, examining where updates are needed and identifying specific mechanics are a priority in order to ensure that decision-making processes in the dental fields are based on the best up-to-date evidence. This finding does not apply completely to Cochrane SRs, given that authors of Cochrane SRs are supposed to update their reports every two years according to Cochrane standards, [6], [19] although a previously published report [13] identified a considerable portion (38%) of the Cochrane child-related SRs as not up-to-date based on the Cochrane criteria.

The results showed that 78 (7.3%) of the non-Cochrane SRs were single-authored, while nearly half of the SRs involved authors from multiple locations and included four to six authors. Having at least two assessors to select relevant reports and extract data in duplicate reduces the potential selection and extraction bias and decreases the possibility of accidental exclusion of relevant reports and inaccurate extraction of relevant data, which may lead to distorted conclusions. [20]–[22] In addition, only one or two databases were searched by approximately half of the non-Cochrane SRs. This is problematic because failure to search multiple databases may lead to missing relevant studies, which can produce biased results and possibly mislead decision-making related to dental practice. [23]–[26].

The results also revealed that the research design of the included studies varied across dental specialties and by type of the SR. While almost all the Cochrane SRs included RCTs only, a small proportion (17.6%) of the non-Cochrane SRs exclusively included RCTs. This may be attributed to Cochrane policy and guidance, which has historically focused on reviews of health care interventions and inclusion of only RCT. This policy explains why all the retrieved Cochrane SRs were therapeutic, while only 72% of the non-Cochrane SRs were therapeutic. Moreover, the nature of the interventions varied across the dental specialties, with nearly two-thirds of all the therapeutic SRs examining non-drug related interventions. This proportion is higher than the proportion found in previous reports examined in medical SRs, [5], [13] and possibly reflects the greater variability in oral health interventions compared to medical interventions. Interestingly, a sizable proportion of the Cochrane SRs (17.5%), including nearly a third of the SRs in the field of oral and maxillofacial surgery, found no appropriate trials to be included. This may be explained by Cochrane’s selective policy of only including RCTs in study selection, considering MAs of RCTs with low risk of bias as the highest level of evidence on the efficacy of treatment interventions. [2] This proportion is higher than the proportion of child-related Cochrane SRs (9.3%) found by Bow et al [13], and possibly highlights the need for more trials to be conducted in the dental specialties, specifically related to oral and maxilla-facial surgery. Similarly, the number of included studies varied across dental specialties and by type of SR. The median number of studies included in Cochrane SRs was five, ranging from two in oral medicine and oral pathology to 12 for dental public health. This median number is less than the number found in child-related Cochrane SRs (seven studies), [13] and again reflects a clear need for more studies to be conducted in the dental specialties.

### Strengths and Limitations

This cross-sectional observational study provides a comprehensive descriptive analysis of all SRs published in the domain of oral health research from inception through May 2012. Our data searches covered six different databases in addition to the ADA-Evidence-based Dentistry website [10], which contains a list of systematic/literature reviews related to oral health research. The addition of this website in our search complemented the other databases searched, making it more comprehensive. However, one of the clear limitations in our research is the data extraction method, which was performed by one assessor. This is problematic because it creates the potential for bias, even though accuracy was assessed by having a 20% random sample (250 SRs) examined in duplicate by two assessors. A further limitation is that we extracted data based on what was reported by the authors of the SRs and, thus, it is possible that some characteristics, such as the type of study included in the SRs, were inappropriately reported by the authors or altogether omitted (which occurred with the source of funding). Another potential limitation is that the implantology-related SRs were categorised in one of three specialties (periodontics, oral and maxillofacial surgery, or prosthodontics), as the ADA classification [11] utilized in our study does not classify “implantology” as an individual specialty. Future methodological studies should consider “implantology” as an individual dental field. Additionally, we may have missed some characteristics in our data extraction such as SR registration which is not very well-known to oral health systematic reviewers. Finally, we may have included SRs in our sample that are not directly related to oral health research but are relevant to dental/oral diseases, such as “orofacial pain in patients receiving cancer therapy” [27].

## Conclusion

We have identified and described a total of 1188 oral health (126 Cochrane and 1062 non-Cochrane) SRs published from 1991 through May 2012, encompassing the nine dental specialties. Epidemiological and descriptive characteristics of the oral health SRs varied across the nine dental specialties and by SR category (Cochrane *vs*. non-Cochrane). There is a clear need for more regular updating of SRs. This includes the examination of where updates are needed and the development of mechanisms to regularly update SRs to ensure that dental practice decision-making is based on up-to-date information. Oral health SRs require improvement with respect to having multiple assessors and searching more than one database. Finally, future methodological studies should consider “implantology” as an individual dental specialty.

## Supporting Information

Appendix S1 Table S1, Search Strategies and Results from Different Electronic Databases; Table S2, Continent and Country of Corresponding Author of Oral Health Systematic Reviews; Table S3, Authors and Affiliation of Oral Health Systematic Reviews; Table S4, Focus and Interventions of Oral Health Systematic Reviews; Table S5, Study Designs of Studies Included in Oral Health Systematic Reviews; Table S6, Number of Included Studies of Oral Health Systematic Reviews; Table S7, Number of Studies Contributed Data to the Largest Meta-Analysis in Oral Health Systematic Reviews.
(DOCX)
