# Supplementary material for: A Descriptive Analysis of Oral Health Systematic Reviews Published 1991–2012: Cross Sectional Study
Source: PLoS One. 2013 Sep 30;8(9):e74545. doi: 10.1371/journal.pone.0074545 (PMC3787021; doi:10.1371/journal.pone.0074545)
Supplement: Appendix S1 — Table S1, Search Strategies and Results from Different Electronic Databases; Table S2, Continent and Country of Corresponding Author of Oral Health Systematic Reviews; Table S3, Authors and Affiliation of Oral Health Systematic Reviews; Table S4, Focus and Interventions of Oral Health Systematic Reviews; Table S5, Study Designs of Studies Included in Oral Health Systematic Reviews; Table S6, Number of Included Studies of Oral Health Systematic Reviews; Table S7, Number of Studies Contributed Data to the Largest Meta-Analysis in Oral Health Systematic Reviews. (DOCX) [file pone.0074545.s001.docx]

| ***Table S1. Search Strategies and Results from Different Electronic Databases*** | | |
| --- | --- | --- |
| **Database** | **Search Strategy** | **Results** |
| PubMed | ((systematic review* OR meta-analys*)) AND (dent* OR tooth OR teeth OR orthodon* OR oral surg* OR endodon* OR periodon* OR prosthodon* OR pedodon* OR pediatric* AND dentistry OR paediatric* AND dentistry OR dent* AND public health OR oral pathology) | 1505 |
| Embase | (systematic review* or meta-analys*).mp. [mp=ti, ab, sh, hw, tn, ot, dm, mf, dv, kw, nm, ps, rs, ui] AND (dent* OR tooth OR teeth OR orthodon* OR oral surg* OR endodon* OR periodon* OR prosthodon* OR pedodon* OR pediatric* dentistry OR paediatric* dentistry OR dent* public health OR oral pathology).mp. [mp=ti, ab, sh, hw, tn, ot, dm, mf, dv, kw, nm, ps, rs, ui] | 2196 |
| MEDLINE (Ovid) | (systematic review* or meta-analys*).mp. [mp=ti, ab, sh, hw, tn, ot, dm, mf, dv, kw, nm, ps, rs, ui] AND (dent* OR tooth OR teeth OR orthodon* OR oral surg* OR endodon* OR periodon* OR prosthodon* OR pedodon* OR pediatric* dentistry OR paediatric* dentistry OR dent* public health OR oral pathology).mp. [mp=ti, ab, sh, hw, tn, ot, dm, mf, dv, kw, nm, ps, rs, ui] | 1709 |
| ISI Web of Science | Topic=(dent* OR tooth OR teeth OR orthodon* OR oral surg* OR endodon* OR periodon* OR prosthodon* OR pedodon* OR pediatric* AND dentistry OR paediatric* AND dentistry OR dent* AND public health OR oral pathology) AND Topic=(systematic review* OR meta-analys*) | 1872 |
| EMB Reviews-Cochrane Database of Systematic Reviews | (systematic review* or meta-analys*).mp. [mp=ti, ab, sh, hw, tn, ot, dm, mf, dv, kw, nm, ps, rs, ui] AND (dent* OR tooth OR teeth OR orthodon* OR oral surg* OR endodon* OR periodon* OR prosthodon* OR pedodon* OR pediatric* dentistry OR paediatric* dentistry OR dent* public health OR oral pathology).mp. [mp=ti, ab, sh, hw, tn, ot, dm, mf, dv, kw, nm, ps, rs, ui] | 559 |
| HealthSTAR | (systematic review* or meta-analys*).mp. [mp=ti, ab, sh, hw, tn, ot, dm, mf, dv, kw, nm, ps, rs, ui] AND (dent* OR tooth OR teeth OR orthodon* OR oral surg* OR endodon* OR periodon* OR prosthodon* OR pedodon* OR pediatric* dentistry OR paediatric* dentistry OR dent* public health OR oral pathology).mp. [mp=ti, ab, sh, hw, tn, ot, dm, mf, dv, kw, nm, ps, rs, ui] | 1828 |
| Total electronic databases searches |  | 9669 |
| Duplicates |  | 2854 |
| Final |  | 6815 |

| ***Table S2. Continent and Country of Corresponding Author of Oral Health Systematic Reviews*** | | | | | | | | | | | | |
| --- | --- | --- | --- | --- | --- | --- | --- | --- | --- | --- | --- | --- |
|  | **Overall**  (No. Overall=1188: NCRs=1062 & CRs=126) | **Oral Medicine**  **& Oral Pathology**  (No. Overall=162: NCRs=140 & CRs=22) | **Dental Public Health**  (No. Overall=184: NCRs=163 & CRs=21) | | **Prosthodontics & Restorative Dentistry**  (No. Overall=198: NCRs=179 & CRs=19) | | **Pediatric Dentistry**  (No. Overall=50: NCRs=42 & CRs=8) | **Endodontics**  (No. Overall=54: NCRs=47 & CRs=7) | **Periodontics** (No. Overall=212: NCRs=203 & CRs=9) | **Orthodontics &Dentofacial Orthopedics**  (No. Overall=138: NCRs=123 & CRs=15) | **Oral and Maxillofacial**  **Surgery** (No. Overall=159: NCRs=134 & CRs=25) | **Oral and Maxillo-facial Radiology** (No. Overall=31: NCRs=31 & CRs=0) |
| Continent of Corresponding Author, n (% total) | | | | | | | | | | | | |
| ***Overall (Cochrane & Non-Cochrane Reviews)*** | | | | | | | | | | | | |
| *Europe* | 645 (54.3) | 87 (53.7) | 88 (47.8) | | 102 (51.5) | | 22 (44) | 17 (31.5) | 126(59.4) | 81 (58.7) | 106 (66.7) | 16 (51.6) |
| *North America* | 303 (25.5) | 46 (28.4) | 60 (32.6) | | 46 (23.2) | | 4 (8) | 16 (29.6) | 51 (24.1) | 37 (26.8) | 33 (20.8) | 10 (32.3) |
| *Asia* | 99 (8.3) | 19 (11.7) | 7 (3.8) | | 15 (7.6) | | 5 (10) | 16 (29.6) | 12 (5.7) | 10 (7.5) | 14 (8.8) | 1 (3.2) |
| *South America* | 61 (5.1) | 4 (2.5) | 6 (3.3) | | 11 (5.6) | | 8 (16) | 2 (3.7) | 17 (8.0) | 7 (5.1) | 4 (2.5) | 2 (6.5) |
| *Australia* | 47 (4.0) | 2 (1.2) | 8 (4.3) | | 17 (8.6) | | 9 (18) | 3 (5.6) | 6 (2.8) | 0 (0.0) | 1 (0.6) | 1 (3.2) |
| *Africa* | 33 (2.8) | 4 (2.5) | 15 (8.2) | | 7 (3.5) | | 2 (4) | 0 (0.0) | (0.0) | 3 (2.2) | 1 (0.6) | 1 (3.2) |
| ***Cochrane Reviews*** | | | | | | | | | | | | |
| *Europe* | 99 (78.6) | 14 (63.6) | 20 (95.2) | | 10 (52.6) | | 6 (75.0) | 5 (71.4) | 8 (88.9) | 12 (80.0) | 24 (96) | 0 (0.0) |
| *North America* | 2 (1.6) | 0 (0.0) | 0 (0.0) | | 2 (10.5) | | 0 (0.0) | 0 (0.0) | 0 (0.0) | 0 (0.0) | 0 (0.0) | 0 (0.0) |
| *Asia* | 13 (10.3) | 6 (27.3) | 1 (4.8) | | 2 (10.5) | | 1 (12.5) | 1 (14.3) | 0 (0.0) | 1 (6.7) | 1 (4) | 0 (0.0) |
| *South America* | 10 (7.9) | 1 (4.5) | 0 (0.0) | | 5 (26.3) | | 0 (0.0) | 1 (14.3) | 1 (11.1) | 2 (13.3) | 0 (0.0) | 0 (0.0) |
| *Australia* | 0 (0.0) | 0 (0.0) | 0 (0.0) | | 0 (0.0) | | 0 (0.0) | 0 (0.0) | 0 (0.0) | 0 (0.0) | 0 (0.0) | 0 (0.0) |
| *Africa* | 2 (1.6) | 1 (4.5) | 0 (0.0) | | 0 (0.0) | | 1 (12.5) | 0 (0.0) | 0 (0.0) | 0 (0.0) | 0 (0.0) | 0 (0.0) |
| ***Non-Cochrane Reviews*** | | | | | | | | | | | | |
| *Europe* | 546 (51.4) | 73 (52.1) | 68 (41.7) | | 92 (51.4) | | 16 (38.1) | 12 (25.5) | 118 (58.1) | 69 (56.1) | 82 (61.2) | 16 (51.6) |
| *North America* | 301 (28.3) | 46 (32.9) | 60 (36.8) | | 44 (24.6) | | 4 (9.5) | 16 (34) | 51 (25.1) | 37 (30.1) | 33 (24.6) | 10 (32.3) |
| *Asia* | 86 (8.1) | 13 (9.3) | 6 (3.7) | | 13 (7.3) | | 4 (9.5) | 15 (31.9) | 12 (5.9) | 9 (7.3) | 13 (9.7) | 1 (3.2) |
| *South America* | 51 (4.8) | 3 (2.1) | 6 (3.75) | | 6 (3.4) | | 8 (19) | 1 (2.1) | 16 (7.9) | 5 (4.1) | 4 (3) | 2 (6.5) |
| *Australia* | 47 (4.4) | 2 (1.4) | 8 (4.9) | | 17 (9.5) | | 9 (21.4) | 3 (6.4) | 6 (3) | 0 (0.0) | 1 (0.7) | 1 (3.2) |
| *Africa* | 31 (2.9) | 3 (2.1) | 15 (9.2) | | 7 (3.9) | | 1 (2.4) | 0 (0.0) | 0 (0.0) | 3 (2.4) | 1 (0.7) | 1 (3.2) |
| Country of Corresponding Author, n (% total) | | | | | | | | | | | | |
| ***Overall (Cochrane & Non-Cochrane Reviews)*** | | | | | | | | | | | | |
| *No. of countries* | 47 | 25 | | 22 | | 29 | 17 | 18 | 29 | 22 | 23 | 14 |
| *USA* | 218 (18.4) | 31 (19.1) | | 46 (25) | | 33 (16.7) | 4 (8.0) | 15 (27.8) | 46 (21.7) | 12 (8.7) | 27 (17) | 4 (12.9) |
| *UK* | 196 (16.5) | 33 (20.4) | | 37 (20.1) | | 15 (7.6) | 14 (28) | 8 (14.8) | 24 (11.3) | 27 (19.6) | 36 (22.6) | 2 (6.5) |
| *Canada* | 85 (7.2) | 15 (9.3) | | 14 (7.6) | | 13 (6.6) | 0 (0.0) | 1 (1.9) | 5 (2.4) | 25 (18.1) | 6 (3.8) | 6 (19.4) |
| *The Netherlands* | 82 (6.9) | 8 (4.9) | | 18 (9.8) | | 12 (6.1) | 1 (2.0) | 0 (0.0) | 14 (6.6) | 11 (8.0) | 14 (8.8) | 4 (12.9) |
| *Switzerland* | 67 (5.6) | 4 (2.5) | | 4 (2.2) | | 24 (12.1) | 1 (2.0) | 1 (1.9) | 16 (7.5) | 5 (3.6) | 12 (7.5) | 0 (0.0) |
| *Italy* | 65 (5.5) | 17 (10.5) | | 3 (1.6) | | 5 (2.5) | 0 (0.0) | 4 (7.4) | 14 (6.6) | 9 (6.5) | 12 (7.5) | 1 (3.2) |
| *Brazil* | 57 (4.8) | 3 (1.9) | | 6 (3.3) | | 10 (5.1) | 7 (14.0) | 2 (3.7) | 16 (7.5) | 7 (5.1) | 4 (2.5) | 2 (6.5) |
| *Germany* | 46 (3.9) | 2 (1.2) | | 1 (0.5) | | 18 (9.1) | 1 (2.0) | 0 (0.0) | 13 (6.1) | 2 (1.4) | 8 (5.0) | 1 (3.2) |
| *Sweden* | 40 (3.4) | 6 (3.7) | | 8 (4.3) | | 2 (1.0) | 1 (2.0) | 1 (1.9) | 11 (5.2) | 7 (5.1) | 2 (1.3) | 2 (6.5) |
| *China* | 40 (3.4) | 8 (4.9) | | 5 (2.7) | | 2 (1.0) | 2 (4.0) | 3 (5.6) | 2 (0.9) | 8 (5.8) | 10 (6.3) | 0 (0.0) |
| *Greece* | 28 (2.4) | 1 (0.6) | | 0 (0.0) | | 5 (2.5) | 1 (2.0) | 0 (0.0) | 10 (4.7) | 10 (7.2) | 1 (0.6) | 0 (0.0) |
| *Australia* | 28 (2.4) | 0 (0.0) | | 4 (2.2) | | 8 (4.0) | 9 (18.0) | 3 (5.6) | 3 (1.4) | 0 (0.0) | 1 (0.6) | 0 (0.0) |
| *Spain* | 25 (2.1) | 9 (5.6) | | 1 (0.5) | | 1 (0.5) | 0 (0.0) | 0 (0.0) | 4 (1.9) | 2 (1.4) | 8 (5.0) | 0 (0.0) |
| *South Africa* | 25 (2.1) | 1 (0.6) | | 15 (8.2) | | 7 (3.5) | 1 (2.0) | 0 (0.0) | 0 (0.0) | 1 (0.7) | 0 (0.0) | 0 (0.0) |
| *Denmark* | 19 (1.6) | 2 (1.2) | | 4 (2.2) | | 0 (0.0) | 1 (2.0) | 1 (1.9) | 4 (1.9) | 0 (0.0) | 5 (3.1) | 2 (6.5) |
| *Belgium* | 16 (1.3) | 0 (0.0) | | 1 (0.5) | | 4 (2.0) | 0 (0.0) | 0 (0.0) | 5 (2.4) | 2 (1.4) | 2 (1.3) | 2 (6.5) |
| *New Zealand* | 16 (1.3) | 1 (0.6) | | 2 (1.1) | | 9 (4.5) | 0 (0.0) | 0 (0.0) | 3 (1.4) | 0 (0.0) | 0 (0.0) | 1 (3.2) |
| *Japan* | 16 (1.3) | 2 (1.2) | | 0 (0.0) | | 10 (5.1) | 1 (2.0) | 1 (1.9) | 1 (0.5) | 0 (0.0) | 1 (0.6) | 0 (0.0) |
| *Other* | 119 (10) | 19 (11.7) | | 15 (8.2) | | 20 (10.1) | 6 (12.0) | 14 (25.9) | 21 (9.9) | 10 (7.2) | 10 (6.3) | 4 (12.9) |
| ***Cochrane Reviews*** | | | | | | | | | | | | |
| *No. of countries* | 20 | 8 | | 5 | | 9 | 3 | 4 | 3 | 3 | 5 | 0 |
| *UK* | 82 (65.1) | 12 (54.5) | | 16 (76.2) | | 7 (36.8) | 6 (75) | 2 (28.6) | 7 (77.8) | 12 (80.0) | 20 (80) | 0 (0.0) |
| *Brazil* | 9 (7.1) | 0 (0.0) | | 0 (0.0) | | 5 (26.3) | 0 (0.0) | 1 (14.3) | 1 (11.1) | 2 (13.3) | 0 (0.0) | 0 (0.0) |
| *Bahrain* | 6 (4.8) | 3 (13.6) | | 0 (0.0) | | 1 (5.3) | 0 (0.0) | 1 (14.3) | 0 (0.0) | 0 (0.0) | 1 (4.0) | 0 (0.0) |
| *China* | 5 (4.0) | 2 (9.1) | | 1 (4.8) | | 0 (0.0) | 0 (0.0) | 0 (0.0) | 0 (0.0) | 1 (6.7) | 1 (4.0) | 0 (0.0) |
| *Germany* | 4 (3.2) | 1 (4.5) | | 0 (0.0) | | 0 (0.0) | 0 (0.0) | 0 (0.0) | 1 (11.1) | 0 (0.0) | 2 (8.0) | 0 (0.0) |
| *Italy* | 4 (3.2) | 1 (4.5) | | 0 (0.0) | | 0 (0.0) | 0 (0.0) | 3 (42.9) | 0 (0.0) | 0 (0.0) | 0 (0.0) | 0 (0.0) |
| *France* | 2 (1.6) | 0 (0.0) | | 1 (4.8) | | 1 (5.3) | 0 (0.0) | 0 (0.0) | 0 (0.0) | 0 (0.0) | 0 (0.0) | 0 (0.0) |
| *Finland* | 2 (1.6) | 0 (0.0) | | 2 (9.5) | | 0 (0.0) | 0 (0.0) | 0 (0.0) | 0 (0.0) | 0 (0.0) | 0 (0.0) | 0 (0.0) |
| *Other* | 12 (9.5) | 3 (13.6) | | 1 (4.8) | | 5 (26.3) | 2 (25) | 0 (0.0) | 0 (0.0) | 0 (0.0) | 1 (4.0) | 0 (0.0) |
| ***Non-Cochrane Reviews*** | | | | | | | | | | | | |
| *No. of countries* | 47 | 22 | | 22 | | 26 | 17 | 18 | 29 | 22 | 23 | 14 |
| *USA* | 217 (20.4) | 31 (22.5) | | 46 (28.2) | | 32 (17.9) | 4 (9.5) | 15 (31.9) | 46 (22.7) | 12 (9.8) | 27 (20.1) | 4 (12.9) |
| *UK* | 114 (10.7) | 21 (15) | | 21 (12.9) | | 8 (4.5) | 8 (19) | 6 (12.8) | 17 (8.4) | 15 (12.2) | 16 (11.9) | 2 (6.5) |
| *Canada* | 84 (7.9) | 15 (10.7) | | 14 (8.6) | | 12 (6.7) | 0 (0.0) | 1 (2.1) | 5 (2.5) | 25 (20.3) | 6 (4.5) | 6 (19.4) |
| *The Netherlands* | 81 (7.6) | 8 (5.7) | | 18 (11.0) | | 12 (6.7) | 1 (2.4) | 0 (0.0) | 14 (6.9) | 11 (8.9) | 13 (9.7) | 4 (12.9) |
| *Switzerland* | 67 (6.3) | 4 (2.9) | | 4 (2.5) | | 24 (13.4) | 1 (2.4) | 1 (2.1) | 16 (7.9) | 5 (4.1) | 12 (9.0) | 0 (0.0) |
| *Italy* | 61 (5.7) | 16 (11.4) | | 3 (1.8) | | 5 (2.8) | 0 (0.0) | 1 (2.1) | 14 (6.9) | 9 (7.3) | 12 (9.0) | 1 (3.2) |
| *Brazil* | 48 (4.5) | 3 (2.1) | | 6 (3.7) | | 5 (2.8) | 7 (16.7) | 1 (2.1) | 15 (7.4) | 5 (4.1) | 4 (3.0) | 2 (6.5) |
| *Germany* | 42 (4.0) | 1 (0.7) | | 1 (0.6) | | 18 (10.1) | 1 (2.4) | 0 (0.0) | 12 (5.9) | 2 (1.6) | 6 (4.5) | 1 (3.2) |
| *Sweden* | 40 (3.8) | 6 (4.3) | | 8 (4.9) | | 2 (1.1) | 1 (2.4) | 1 (2.1) | 11 (5.4) | 7 (5.7) | 2 (1.5) | 2 (6.5) |
| *Greece* | 28 (2.6) | 1 (0.7) | | 0 (0.0) | | 5 (2.8) | 1 (2.4) | 0 (0.0) | 10 (4.9) | 10 (8.1) | 1 (0.7) | 0 (0.0) |
| *Australia* | 28 (2.6) | 0 (0.0) | | 4 (2.5) | | 8 (4.5) | 9 (21.4) | 3 (6.4) | 3 (1.5) | 0 (0.0) | 1 (0.7) | 0 (0.0) |
| *Other* | 252 (23.7) | 34 (24.3) | | 38 (23.3) | | 48 (26.8) | 9 (21.4) | 18 (38.3) | 40 (19.7) | 22 (17.9) | 34 (25.4) | 9 (29) |

| ***Table S3. Authors and Affiliation of Oral Health Systematic Reviews*** | | | | | | | | | | | | | | | | |
| --- | --- | --- | --- | --- | --- | --- | --- | --- | --- | --- | --- | --- | --- | --- | --- | --- |
|  | **Overall**  (No. Overall=1188: NCRs=1062 & CRs=126) | **Oral Medicine**  **& Oral Pathology**  (No. Overall=162: NCRs=140 & CRs=22) | **Dental Public Health**  (No. Overall=184: NCRs=163 & CRs=21) | **Prosthodontics & Restorative Dentistry**  (No. Overall=198: NCRs=179 & CRs=19) | | **Pediatric Dentistry**  (No. Overall=50: NCRs=42 & CRs=8) | | **Endodontics**  (No. Overall=54: NCRs=47 & CRs=7) | | **Periodontics** (No. Overall=212: NCRs=203 & CRs=9) | | **Orthodontics &Dentofacial Orthopedics**  (No. Overall=138: NCRs=123 & CRs=15) | | **Oral and Maxillofacial**  **Surgery** (No. Overall=159: NCRs=134 & CRs=25) | | **Oral and Maxillo-facial Radiology** (No. Overall=31: NCRs=31 & CRs=0) |
| Number of Authors | | | | | | | | | | | | | | | | |
| ***Overall (Cochrane & Non-Cochrane Reviews)*** | | | | | | | | | | | | | | | | |
| **Number of authors, median (IQR)** | | | | | | | | | | | | | | | | |
|  | 4 (2, 5) | 4 (3, 6) | 3 (2, 4.75) | 3 (2, 5) | | 3 (2, 4) | | 4 (3, 5) | | 3.5  (2.25, 5) | | 3 (3, 5) | | 4 (3, 5) | | 3.5  (2.25, 4.75) |
| **Number of authors, n (% total)** | | | | | | | | | | | | | | | | |
| 1 | 78 (6.6) | 13 (8.0) | 18 (9.8) | 10 (5.1) | | 6 (12) | | 0 (0.0) | | 13 (6.1) | | 6 (4.3) | | 7 (4.4) | | 5 (16.1) |
| 2-3 | 505 (42.5) | 55 (34) | 83 (45.1) | 90 (45.5) | | 20 (40) | | 23 (42.6) | | 93 (43.9) | | 66 (47.8) | | 64 (40.3) | | 11 (35.5) |
| 4-6 | 520 (43.8) | 72 (44.4) | 65 (35.3) | 88 (44.4) | | 19 (38) | | 29 (53.7) | | 98 (46.2) | | 58 (42.0) | | 77 (48.4) | | 14 (45.2) |
| ≥ 7 | 85 (7.2) | 22 (13.6) | 18 (9.8) | 10 (5.1) | | 5 (10) | | 2 (3.7) | | 8 (3.8) | | 8 (5.8) | | 11 (6.9) | | 1 (3.2) |
| ***Cochrane Reviews*** | | | | | | | | | | | | | | | | |
| **Number of authors, median (IQR)** | | | | | | | | | | | | | | | | |
|  | 5 (4, 6) | 4.5 (3.75,6) | 4 (4, 6.5) | 4 (3, 6) | | 4 (3,4.75) | | 5 (4, 5) | | 5 (4, 5) | | 5 (3, 6) | | 5 (4, 6.75) | | N/A |
| **Number of authors, n (% total)** | | | | | | | | | | | | | | | | |
| 1 | 0 (0.0) | 0 (0.0) | 0 (0.0) | 0 (0.0) | | 0 (0.0) | | 0 (0.0) | | 0 (0.0) | | 0 (0.0) | | 0 (0.0) | | 0 (0.0) |
| 2-3 | 26 (20.6) | 5 (22.7) | 1 (4.8) | 6 (31.5) | | 3 (37.5) | | 0 (0.0) | | 1 (11.1) | | 5 (33.3) | | 5 (20.0) | | 0 (0.0) |
| 4-6 | 81 (64.3) | 15 (68.2) | 15 (71.4) | 11 (57.9) | | 5 (62.5) | | 7 (100) | | 8 (88.9) | | 7 (46.7) | | 13 (52.0) | | 0 (0.0) |
| ≥ 7 | 19 (15.1) | 2 (9.1) | 5 (23.8) | 2 (10.5) | | 0 (0.0) | | 0 (0.0) | | 0 (0.0) | | 3 (20.0) | | 7 (28.0) | | 0 (0.0) |
| ***Non-Cochrane Reviews*** | | | | | | | | | | | | | | | | |
| **Number of authors, median (IQR)** | | | | | | | | | | | | | | | | |
|  | 3 (2, 5) | 4 (3, 5.75) | 3 (2, 4) | 3 (2, 4) | | 2.5  (2, 4.25) | | 4 (3, 5) | | 3 (2, 5) | | 3 (2, 5) | | 4 (2, 5) | | 3 (2, 4) |
| **Number of authors, n (% total)** | | | | | | | | | | | | | | | | |
| 1 | 78 (7.3) | 13 (9.3) | 18 (11.0) | 10 (5.6) | | 6 (14.3) | | 0 (0.0) | | 13 (6.4) | | 6 (4.9) | | 7 (5.2) | | 5 (16.1) |
| 2-3 | 479 (45.1) | 50 (35.7) | 82 (50.3) | 84 (46.9) | | 17 (40.5) | | 23 (48.9) | | 92 (45.3) | | 61 (49.6) | | 59 (44.0) | | 11 (35.5) |
| 4-6 | 439 (41.3) | 57 (40.7) | 50 (30.7) | 77 (43.0) | | 14 (33.3) | | 22 (46.8) | | 90 (44.3) | | 51 (41.5) | | 64 (47.8) | | 14 (45.2) |
| ≥ 7 | 66 (6.2) | 20 (14.3) | 13 (80.0) | 8 (4.5) | | 5 (11.9) | | 2 (4.3) | | 8 (3.9) | | 5 (4.1) | | 4 (3.0) | | 1 (3.2) |
| Number of Schools/Affiliations | | | | | | | | | | | | | | | | |
| ***Overall (Cochrane & Non-Cochrane Reviews)*** | | | | | | | | | | | | | | | | |
| **Number of schools, median (IQR)** | | | | | | | | | | | | | | | | |
|  | 2 (1, 3) | 2 (1, 3) | 2 (1, 3) | 2 (1, 3) | 1.5 (1, 2) | | 2 (1, 3) | | 2 (1, 3) | | 2 (1, 2) | | 2 (1, 3) | | 2 (1, 2) | |
| **Number of schools, n (% total)** | | | | | | | | | | | | | | | | |
| 1 | 454 (38.2) | 44 (27.2) | 66 (35.9) | 81 (40.9) | 25 (50) | | 25 (46.3) | | 74 (34.9) | | 60 (43.5) | | 67 (42.1) | | 12 (38.7) | |
| 2-3 | 573 (48.2) | 82 (50.6) | 82 (44.6) | 94 (47.5) | 19 (38) | | 19 (35.2) | | 118(55.7) | | 65 (47.1) | | 77 (48.4) | | 17 (54.8) | |
| 4≤ | 161 (13.6) | 36 (22.2) | 36 (19.6) | 23 (11.6) | 6 (12) | | 10 (18.5) | | 20 (9.4) | | 13 (9.4) | | 15 (9.4) | | 2 (6.5) | |
| ***Cochrane Reviews*** | | | | | | | | | | | | | | | | |
| **Number of schools, median (IQR)** | | | | | | | | | | | | | | | | |
|  | 3 (2, 4) | 3 (2, 4) | 4 (3, 4.5) | 3 (2, 4) | 3 (1.5, 4) | | 4 (2, 4) | | 2  (1.5, 3.5) | | 3 (2, 5) | | 2 (1, 4) | | N/A | |
| **Number of schools, n (% total)** | | | | | | | | | | | | | | | | |
| 1 | 19 (15.1) | 2 (9.1) | 0 (0.0) | 2 (10.5) | 2 (25.0) | | 1 (14.3) | | 2 (22.2) | | 1 (6.7) | | 9 (36.0) | | 0 (0.0) | |
| 2-3 | 57 (45.2) | 13 (59.1) | 7 (33.3) | 11 (57.9) | 3 (37.5) | | 2 (28.6) | | 5 (55.6) | | 8 (53.3) | | 8 (32.0) | | 0 (0.0) | |
| 4≤ | 50 (39.7) | 7 (31.8) | 14 (66.7) | 6 (31.6) | 3 (37.5) | | 4 (57.1) | | 2 (22.2) | | 6 (40.0) | | 8 (32.0) | | 0 (0.0) | |
| ***Non-Cochrane Reviews*** | | | | | | | | | | | | | | | | |
| **Number of schools, median (IQR)** | | | | | | | | | | | | | | | | |
|  | 2 (1, 3) | 2 (1, 3) | 2 (1, 3) | 2 (1, 2) | 1 (1, 2) | | 1 (1, 3) | | 2 (1, 3) | | 2 (1, 2) | | 2 (1, 2) | | 2 (1, 2) | |
| **Number of schools, n (% total)** | | | | | | | | | | | | | | | | |
| 1 | 435 (41.0) | 42 (30.0) | 66 (40.5) | 79 (44.1) | 23 (54.8) | | 24 (51.1) | | 72 (35.5) | | 59 (48) | | 58 (43.3) | | 12 (38.7) | |
| 2-3 | 516 (48.6) | 69 (49.3) | 75 (46.0) | 83 (46.4) | 16 (38.1) | | 17 (36.2) | | 113(55.7) | | 57 (46.3) | | 69 (51.5) | | 17 (54.8) | |
| 4≤ | 111 (10.5) | 29 (20.7) | 22 (13.5) | 17 (19.5) | 3 (7.1) | | 6 (12.8) | | 18 (8.9) | | 7 (5.7) | | 7 (5.2) | | 2 (6.5) | |
| NCRs, non-Cochrane Reviews; CRs, Cochrane Reviews; IQR, interquartile range, N/A, not applicable. | | | | | | | | | | | | | | | | |

| ***Table S4. Focus and Interventions of Oral Health Systematic Reviews*** | | | | | | | | | | | | |
| --- | --- | --- | --- | --- | --- | --- | --- | --- | --- | --- | --- | --- |
|  | **Overall**  (No. Overall=1188: NCRs=1062 & CRs=126) | | **Oral Medicine**  **& Oral Pathology**  (No. Overall=162: NCRs=140 & CRs=22) | **Dental Public Health**  (No. Overall=184: NCRs=163 & CRs=21) | **Prosthodontics & Restorative Dentistry**  (No. Overall=198: NCRs=179 & CRs=19) | **Pediatric Dentistry**  (No. Overall=50: NCRs=42 & CRs=8) | | **Endodontics**  (No. Overall=54: NCRs=47 & CRs=7) | **Periodontics** (No. Overall=212: NCRs=203 & CRs=9) | **Orthodontics &Dentofacial Orthopedics**  (No. Overall=138: NCRs=123 & CRs=15) | **Oral and Maxillofacial**  **Surgery** (No. Overall=159: NCRs=134 & CRs=25) | **Oral and Maxillo-facial Radiology** (No. Overall=31: NCRs=31 & CRs=0) |
| Type of Review, N (% Total) | | | | | | | | | | | | |
| ***Overall (Cochrane & Non-Cochrane Reviews)*** | | | | | | | | | | | | |
| Therapeutic | 894 (75.3) | | 80 (49.4) | 133 (72.3) | 183 (92.4) | 38 (76) | | 48 (88.9) | 151 (71.2) | 113 (81.9) | 146 (91.8) | 2 (6.5) |
| Non-therapeutic | 294 (24.7) | | 82 (50.6) | 51 (27.7) | 15 (7.6) | 12 (24) | | 6 (11.1) | 61 (28.8) | 25 (18.1) | 13 (8.2) | 29 (93.5) |
| ***Cochrane Reviews*** | | | | | | | | | | | | |
| Therapeutic | 126 (100) | | 22 (100) | 21 (100) | 19 (100) | 8 (100) | | 7 (100) | 9 (100) | 15 (100) | 25 (100) | 0 (0.0) |
| Non-therapeutic | 0 (0.0) | | 0 (0.0) | 0 (0.0) | 0 (0.0) | 0 (0.0) | | 0 (0.0) | 0 (0.0) | 0 (0.0) | 0 (0.0) | 0 (0.0) |
| ***Non-Cochrane Reviews*** | | | | | | | | | | | | |
| Therapeutic | 768 (72.3) | | 58 (41.4) | 112 (68.7) | 164 (91.6) | 30 (71.4) | | 41 (87.2) | 142 (70.0) | 98 (79.7) | 121 (90.3) | 2 (6.5) |
| Non-therapeutic | 294 (27.7) | | 82 (58.6) | 51 (31.3) | 15 (8.4) | 12 (28.6) | | 6 (12.8) | 61 (30.0) | 25 (20.3) | 13 (9.7) | 29 (93.5) |
| Focus of Non-therapeutic SRs, N (% Total) | | | | | | | | | | | | |
| ***Overall/Non-Cochrane Reviews*** | | | | | | | | | | | | |
| *Total Number* | | N=294 | N=82 | N=51 | N=15 | N=12 | | N=6 | N=61 | N=25 | N=13 | N=29 |
| Diagnosis/  Prognosis | | 112 (38.1) | 24 (29.3) | 16 (31.4) | 3 (20.0) | 4 (33.3) | | 1 (16.7) | 22 (36.1) | 9 (36.0) | 8 (61.5) | 25 (86.2) |
| Epidemiology | | 150 (51) | 56 (68.3) | 26 (51.0) | 10 (66.7) | 6 (50.0) | | 3 (50.0) | 36 (59.0) | 8 (32.0) | 1 (7.7) | 4 (13.8) |
| Psychological/  Educational/Policy/Quality of studies | | 32 (10.9) | 2 (2.4) | 9 (17.6) | 2 (13.3) | 2 (16.7) | | 2 (33.3) | 3 (4.9) | 8 (32.0) | 4 (30.8) | 0 (0.0) |
| Type of Intervention in Therapeutic SRs, Category I, N (% Total) | | | | | | | | | | | | |
| ***Overall (Cochrane & Non-Cochrane Reviews)*** | | | | | | | | | | | | |
| *Total Number* | N=894 | | N=80 | N=133 | N=183 | N=38 | | N=48 | N=151 | N=113 | N=146 | N=2 |
| Drug | 219 (24.5) | | 45 (56.2) | 74 (55.6) | 10 (5.5) | 10 (26.3) | | 13 (27.1) | 28 (18.5) | 6 (5.3) | 33 (22.6) | 0 (0.0) |
| Non-drug | 577 (64.5) | | 19 (23.8) | 51 (38.3) | 169 (92.3) | 19 (50.0) | | 18 (37.5) | 90 (59.6) | 105 (92.9) | 104 (71.2) | 2 (100) |
| Both | 98 (11.0) | | 16 (20.0) | 8 (6.0) | 4 (2.2) | 9 (23.7) | | 17 (35.4) | 33 (21.9) | 2 (1.8) | 9 (6.2) | 0 (0.0) |
| Type of Intervention in Therapeutic SRs, Category II, N (% Total) | | | | | | | | | | | | |
| ***Overall (Cochrane & Non-Cochrane Reviews)*** | | | | | | | | | | | | |
| *Total Number* | N=894 | | N=80 | N=133 | N=183 | N=38 | | N=48 | N=151 | N=113 | N=146 | N=2 |
| Surgical | 151 (16.9) | | 0 (0.0) | 0 (0.0) | 8 (4.4) | 0 (0.0) | | 3 (6.2) | 41 (27.2) | 8 (7.1) | 91 (62.3) | 0 (0.0) |
| Non-surgical | 651 (72.8) | | 67 (83.8) | 132 (99.2) | 160 (87.4) | 36 (94.7) | | 39 (81.2) | 82 (54.3) | 94 (83.2) | 39 (26.7) | 2 (100) |
| Both | 92 (10.3) | | 13 (16.2) | 1 (0.8) | 15 (8.2) | 2 (5.3) | | 6 (12.5) | 28 (18.5) | 11 (9.7) | 16 (11.0) | 0 (0.0) |
| Type of Intervention in Therapeutic SRs, Category III, N (% Total) | | | | | | | | | | | | |
| ***Overall (Cochrane & Non-Cochrane Reviews)*** | | | | | | | | | | | | |
| *Total Number* | | N=894 | N=80 | N=133 | N=183 | N=38 | N=48 | | N=151 | N=113 | N=146 | N=2 |
| Surgical | | 145 (16.2) | 1 (1.2) | 0 (0.0) | 9 (4.9) | 0 (0.0) | 3 (6.2) | | 39 (25.8) | 7 (6.2) | 86 (58.9) | 0 (0.0) |
| Device | | 163 (18.2) | 0 (0.0) | 0 (0.0) | 83 (45.4) | 3 (7.9) | 1 (2.1) | | 3 (2.0) | 70 (61.9) | 3 (2.1) | 0 (0.0) |
| Drug | | 194 (21.7) | 46 (57.5) | 65 (48.9) | 6 (3.3) | 7 (18.4) | 5(10.4) | | 28 (18.5) | 4 (3.5) | 33 (22.6) | 0 (0.0) |
| Dental Material | | 96 (10.7) | 1 (1.2) | 25 (18.8) | 47 (25.7) | 7 (18.4) | 8 (16.7) | | 0 (0.0) | 7 (6.2) | 1 (0.7) | 0 (0.0) |
| Psychological/  Educational/Policy | | 31 (3.5) | 3 (3.8) | 17 (12.8) | 1 (0.5) | 4 (10.5) | 1 (2.1) | | 2 (1.3) | 2 (1.8) | 1 (0.7) | 0 (0.0) |
| Other | | 105 (11.7) | 11 (13.8) | 18 (13.5) | 16 (8.8) | 7 (18.4) | 14 (29.2) | | 30 (19.9) | 5 (4.4) | 2 (1.4) | 2 (100) |
| Multiple/  Combined | | 160 (17.9) | 18 (22.5) | 8 (6.0) | 21 (11.5) | 10 (26.3) | 16 (33.3) | | 49 (32.5) | 18 (15.9) | 20 (13.7) | 0 (0.0) |
| NCRs, non-Cochrane Reviews; CRs, Cochrane Reviews; N/A, not applicable. | | | | | | | | | | | | |

| ***Table S5.*** ***Study Designs of Studies Included in Oral Health Systematic Reviews*** | | | | | | | | | | | |
| --- | --- | --- | --- | --- | --- | --- | --- | --- | --- | --- | --- |
|  | **Overall**  (No. Overall=1188: NCRs=1062 & CRs=126) | **Oral Medicine**  **& Oral Pathology**  (No. Overall=162: NCRs=140 & CRs=22) | **Dental Public Health**  (No. Overall=184: NCRs=163 & CRs=21) | **Prosthodontics & Restorative Dentistry**  (No. Overall=198: NCRs=179 & CRs=19) | **Pediatric Dentistry**  (No. Overall=50: NCRs=42 & CRs=8) | **Endodontics**  (No. Overall=54: NCRs=47 & CRs=7) | **Periodontics** (No. Overall=212: NCRs=203 & CRs=9) | **Orthodontics &Dentofacial Orthopedics**  (No. Overall=138: NCRs=123 & CRs=15) | **Oral and Maxillofacial**  **Surgery** (No. Overall=159: NCRs=134 & CRs=25) | | **Oral and Maxillo-facial Radiology** (No. Overall=31: NCRs=31 & CRs=0) |
| Study Designs of SRs with Eligible Studies | | | | | | | | | | | |
| ***Overall (Cochrane & Non-Cochrane Reviews)*** | | | | | | | | | | | |
| *Total Number* | N=1163 | N=161 | N=183 | N=193 | N=48 | N=50 | N=212 | N=134 | N=151 | N=31 | |
| RCTs only | 283 (24.3) | 39 (24.2) | 61 (33.3) | 33 (17.1) | 10 (20.8) | 16 (32.0) | 62 (29.2) | 21 (15.7) | 40 (26.5) | 1 (3.2) | |
| CCTs only | 10 (0.9) | 0 (0.0) | 0 (0.0) | 3 (1.6) | 0 (0.0) | 0 (0.0) | 1 (0.5) | 2 (1.5) | 4 (2.6) | 0 (0.0) | |
| RCTs and CCTs | 71 (6.1) | 5 (3.1) | 18 (9.8) | 11 (5.7) | 4 (8.3) | 0 (0.0) | 9 (4.2) | 15 (11.2) | 9 (6.0) | 0 (0.0) | |
| RCTs and other designs | 326 (28.0) | 31 (19.3) | 46 (25.1) | 76 (39.4) | 13 (27.1) | 20 (40.0) | 59 (27.8) | 30 (22.4) | 46 (30.5) | 5 (16.1) | |
| Non-RCTs | 424 (36.5) | 84 (52.2) | 52 (28.4) | 54 (28.0) | 21 (43.8) | 12 (24.0) | 72 (34) | 61 (45.5) | 43 (28.5) | 25 (80.6) | |
| Unclear/Not reported | 49 (4.2) | 2 (1.2) | 6 (3.3) | 16 (8.3) | 0 (0.0) | 2 (4.0) | 9 (4.2) | 5 (3.7) | 9 (6.0) | 0 (0.0) | |
| ***Cochrane Reviews*** | | | | | | | | | | | |
| *Total Number* | N=104 | N=21 | N=20 | N=14 | N=6 | N=5 | N=9 | N=11 | N=18 | N=0 | |
| RCTs only | 97 (93.3) | 19 (90.5) | 19 (95) | 13 (92.9) | 6 (100) | 5 (100) | 9 (100) | 8 (72.7) | 18 (100) | 0 (0.0) | |
| CCTs only | 1 (1.0) | 0 (0.0) | 0 (0.0) | 0 (0.0) | 0 (0.0) | 0 (0.0) | 0 (0.0) | 1 (9.1) | 0 (0.0) | 0 (0.0) | |
| RCTs and CCTs | 4 (3.8) | 1 (4.8) | 0 (0.0) | 1 (7.1) | 0 (0.0) | 0 (0.0) | 0 (0.0) | 2 (18.2) | 0 (0.0) | 0 (0.0) | |
| RCTs and other designs | 1 (1.0) | 0 (0.0) | 1 (5) | 0 (0.0) | 0 (0.0) | 0 (0.0) | 0 (0.0) | 0 (0.0) | 0 (0.0) | 0 (0.0) | |
| Non-RCTs | 1 (1.0) | 1 (4.8) | 0 (0.0) | 0 (0.0) | 0 (0.0) | 0 (0.0) | 0 (0.0) | 0 (0.0) | 0 (0.0) | 0 (0.0) | |
| Unclear/Not reported | 0 (0.0) | 0 (0.0) | 0 (0.0) | 0 (0.0) | 0 (0.0) | 0 (0.0) | 0 (0.0) | 0 (0.0) | 0 (0.0) | 0 (0.0) | |
| ***Non-Cochrane Reviews*** | | | | | | | | | | | |
| *Total Number* | N=1059 | N=140 | N=163 | N=179 | N=42 | N=45 | N=203 | N=123 | N=133 | N=31 | |
| RCTs only | 186 (17.6) | 20 (14.3) | 42 (25.8) | 20 (11.2) | 4 (9.5) | 11 (24.4) | 53 (26.1) | 13 (10.6) | 22 (16.5) | 1 (3.2) | |
| CCTs only | 9 (0.8) | 0 (0.0) | 0 (0.0) | 3 (1.7) | 0 (0.0) | 0 (0.0) | 1 (0.5) | 1 (0.8) | 4 (3.0) | 0 (0.0) | |
| RCTs and CCTs | 67 (6.3) | 4 (2.9) | 18 (11.0) | 10 (5.6) | 4 (9.5) | 0 (0.0) | 9 (4.4) | 13 (10.6) | 9 (6.8) | 0 (0.0) | |
| RCTs and other designs | 325 (30.7) | 31 (22.1) | 45 (27.6) | 76 (42.5) | 13 (31.0) | 20 (44.4) | 59 (29.1) | 30 (24.4) | 46 (34.6) | 5 (16.1) | |
| Non-RCTs | 423 (39.9) | 83 (59.3) | 52 (31.9) | 54 (30.2) | 21 (50.0) | 12 (26.7) | 72 (35.5) | 61 (49.6) | 43 (32.3) | 25 (80.6) | |
| Unclear/Not reported | 49 (4.6) | 2 (1.4) | 6 (3.7) | 16 (8.9) | 0 (0.0) | 2 (4.4) | 9 (4.4) | 5 (4.1) | 9 (6.8) | 0 (0.0) | |
| NCRs, non-Cochrane reviews; CRs, Cochrane reviews; RCTs, randomized controlled trials; CCTs, controlled clinical trials. | | | | | | | | | | | |

| ***Table S6. Number of Included Studies of Oral Health Systematic Reviews*** | | | | | | | | | | | | | | | | |
| --- | --- | --- | --- | --- | --- | --- | --- | --- | --- | --- | --- | --- | --- | --- | --- | --- |
|  | **Overall**  (No. Overall=1188: NCRs=1062 & CRs=126) | **Oral Medicine**  **& Oral Pathology**  (No. Overall=162: NCRs=140 & CRs=22) | **Dental Public Health**  (No. Overall=184: NCRs=163 & CRs=21) | | **Prosthodontics & Restorative Dentistry**  (No. Overall=198: NCRs=179 & CRs=19) | | | **Pediatric Dentistry**  (No. Overall=50: NCRs=42 & CRs=8) | **Endodontics**  (No. Overall=54: NCRs=47 & CRs=7) | **Periodontics** (No. Overall=212: NCRs=203 & CRs=9) | | **Orthodontics &Dentofacial Orthopedics**  (No. Overall=138: NCRs=123 & CRs=15) | | **Oral and Maxillofacial**  **Surgery** (No. Overall=159: NCRs=134 & CRs=25) | | **Oral and Maxillo-facial Radiology** (No. Overall=31: NCRs=31 & CRs=0) |
| Number of Included Studies | | | | | | | | | | | | | | | | |
| ***Overall (Cochrane & Non-Cochrane Reviews)*** | | | | | | | | | | | | | | | | |
| **Number of included studies, median (IQR)** | | | | | | | | | | | | | | | | |
|  | 14 (7, 28) | 16.5 (9, 31) | 16 (8, 33) | | 13 (8, 27) | | | 12 (5.5, 25.5) | 11 (4, 30) | 15 (9, 28) | | 11 (5.75, 18.25) | | 13 (7, 25) | | 15.5 (7,28) |
| **Number of included studies, n ( total)** | | | | | | | | | | | | | | | | |
| 0 | 25 (2.1) | 1 (0.6) | 1 (0.5) | | 5 (2.5) | | | 2 (4.0) | 4 (7.4) | 0 (0.0) | | 4 (2.9) | | 8 (5.0) | | 0 (0.0) |
| 1-5 | 166 (14.0) | 21 (13.0) | 16 (8.7) | | 25 (12.6) | | | 10 (20.0) | 12 (22.2) | 25 (11.8) | | 29 (21.0) | | 24 (15.1) | | 4 (12.9) |
| 6-15 | 433 (36.4) | 49 (30.2) | 70 (38.0) | | 69 (34.8) | | | 20 (40.0) | 15 (27.8) | 81 (38.2) | | 61 (44.2) | | 57 (35.8) | | 11 (35.5) |
| 16-30 | 261 (22.0) | 42 (25.9) | 42 (22.8) | | 44 (22.2) | | | 8 (16.0) | 9 (16.7) | 52 (24.5) | | 22 (15.9) | | 34 (21.4) | | 8 (25.8) |
| >30 | 251 (21.1) | 39 (24.1) | 50 (27.2) | | 40 (20.2) | | | 9 (18.0) | 13 (24.1) | 45 (21.2) | | 18 (13.0) | | 30 (18.9) | | 7 (22.6) |
| Unclear/Not reported | 52 (4.4) | 10 (6.2) | 5 (2.7) | | 15 (7.6) | | | 1 (2.0) | 1 (1.9) | 9 (4.2) | | 4 (2.9) | | 6 (3.8) | | 1 (3.2) |
| **Number of included RCTs, median (IQR)** | | | | | | | | | | | | | | | | |
|  | 1 (0, 7) | 0 (0, 6) | 4 (0, 11.5) | | 1 (0, 6) | | | 0 (0, 5) | 3 (0, 6) | 3 (0, 10) | | 0 (0, 4) | | 1 (0, 7) | | 0 (0, 0) |
| **Number of included RCTs, n (% total)** | | | | | | | | | | | | | | | | |
| 0 | 461 (38.3) | 85 (52.5) | 53 (28.8) | | 74 (34.9) | | | 23 (46.0) | 16 (29.6) | 23 (46.0) | 68 (49.3) | | 55 (34.6) | | 25 (80.6) | |
| 1-2 | 116 (9.8) | 14 (8.6) | 14 (7.6) | | 9 (4.2) | | | 3 (6.0) | 7 (13.0) | 3 (6.0) | 17 (12.3) | | 25 (15.7) | | 2 (6.5) | |
| 3-4 | 72 (6.1) | 8 (4.9) | 10 (5.4) | | 19 (9.0) | | | 5 (10.0) | 9 (16.7) | 5 (10.0) | 6 (4.3) | | 8 (5.0) | | 0 (0.0) | |
| 5-10 | 183 (15.4) | 16 (9.9) | 34 (18.5) | | 39 (18.4) | | | 7 (14.0) | 13 (24.1) | 7 (14.0) | 17 (12.3) | | 22 (13.8) | | 0 (0.0) | |
| 11-20 | 96 (8.1) | 15 (9.3) | 26 (14.1) | | 13 (6.1) | | | 4 (8.0) | 3 (5.6) | 4 (8.0) | 10 (7.2) | | 14 (8.8) | | 1 (3.2) | |
| >20 | 75 (75) | 12 (7.4) | 16 (8.7) | | 27 (12.7) | | | 3 (6.0) | 0 (0.0) | 3 (6.0) | 0 (0.0) | | 11 (6.9) | | 0 (0.0) | |
| Unclear/Not reported | 185 (15.6) | 12 (7.4) | 31 (16.8) | | 31 (14.6) | | | 5 (10.0) | 6 (11.1) | 5 (10.0) | 20 (14.5) | | 24 (15.1) | | 3 (9.7) | |
| ***Cochrane Reviews*** | | | | | | | | | | | | | | | | |
| **Number of included studies, median (IQR)** | | | | | | | | | | | | | | | | |
|  | 5 (1, 13) | 9 (2, 19.75) | 12 (3, 30.5) | | 2 (0, 6) | | | 3 (0.75, 10.25) | 3 (0, 4) | 7 (3.5, 15) | 3 (0, 8) | | 2 (0, 12) | | N/A | |
| **Number of included studies, n (% total)** | | | | | | | | | | | | | | | | |
| 0 | 22 (17.5) | 1 (4.5) | 1 (4.8) | | 5 (26.3) | | 2 (25.0) | | 2 (28.6) | 0 (0.0) | 4 (26.7) | | 7 (29.2) | | 0 (0.0) | |
| 1-5 | 45 (35.7) | 8 (36.4) | 5 (23.8) | | 8 (42.1) | | 4 (50.0) | | 4 (57.1) | 3 (33.3) | 6 (40.0) | | 7 (29.2) | | 0 (0.0) | |
| 6-15 | 32 (25.4) | 6 (27.3) | 5 (23.8) | | 4 (21.1) | | 1 (12.5) | | 1 (14.3) | 4 (44.4) | 5 (33.3) | | 6 (24.0) | | 0 (0.0) | |
| 16-30 | 17 (13.5) | 4 (18.2) | 5 (23.8) | | 2 (10.5) | | 0 (0.0) | | 0 (0.0) | 2 (22.2) | 0 (0.0) | | 4 (16.0 ) | | 0 (0.0) | |
| >30 | 10 (7.9) | 3 (13.6) | 5 (23.8) | | 0 (0.0) | | 1 (12.5) | | 0 (0.0) | 0 (0.0) | 0 (0.0) | | 1 (4.0) | | 0 (0.0) | |
| Unclear/Not reported | 0 (0.0) | 0 (0.0) | 0 (0.0) | | 0 (0.0) | | 0 (0.0) | | 0 (0.0) | 0 (0.0) | 0 (0.0) | | 0 (0.0) | | 0 (0.0) | |
| **Number of included RCTs, median (IQR)** | | | | | | | | | | | | | | | | |
|  | 5 (1, 12) | 8.5 (2, 19.75) | 12 (2, 30.5) | | 2 (0, 6) | | 3 (0.75, 10.25) | | 3 (0, 4) | 7 (3.5, 15) | 2 (0, 5) | | 2 (0, 12) | | N/A | |
| **Number of included RCTs, n (% total)** | | | | | | | | | | | | | | | | |
| 0 | 24 (19) | 2 (9.1) | 1 (4.8) | | 5 (26.3) | 2 (25.0) | | | 2 (28.6) | 0 (0.0) | 5 (33.3) | | 7 (28.0) | | 0 (0.0) | |
| 1-2 | 27 (21.4) | 5 (22.7) | 5 (23.8) | | 6 (31.6) | 0 (0.0) | | | 1 (14.3) | 1 (11.1) | 3 (20.0) | | 6 (24.0) | | 0 (0.0) | |
| 3-4 | 11 (8.7) | 1 (4.5) | 1 (4.8) | | 0 (0.0) | 3 (37.5) | | | 3 (42.9) | 2 (22.2) | 1 (6.7) | | 0 (0.0) | | 0 (0.0) | |
| 5-10 | 27 (21.4) | 6 (27.3) | 2 (9.5) | | 5 (26.3) | 1 (12.5) | | | 0 (0.0) | 3 (33.3) | 5 (33.3) | | 5 (20.0) | | 0 (0.0) | |
| 11-20 | 18 (14.3) | 3 (13.6) | 6 (28.6) | | 1 (5.3) | 1 (12.5) | | | 1 (14.3) | 2 (22.2) | 1 (6.7) | | 3 (12.0) | | 0 (0.0) | |
| >20 | 19 (15.1) | 5 (22.7) | 6 (28.6) | | 2 (10.5) | 1 (12.5) | | | 0 (0.0) | 1 (11.1) | 0 (0.0) | | 4 (16.0) | | 0 (0.0) | |
| Unclear/Not reported | 0 (0.0) | 0 (0.0) | 0 (0.0) | | 0 (0.0) | 0 (0.0) | | | 0 (0.0) | 0 (0.0) | 0 (0.0) | | 0 (0.0) | | 0 (0.0) | |
| ***Non-Cochrane Reviews*** | | | | | | | | | | | | | | | | |
| **Number of included studies, median (IQR)** | | | | | | | | | | | | | | | | |
|  | 15 (8, 29) | 17.5  (10, 34.5) | 16.5 (8, 33.25) | 15.5 (9, 29.75) | | 14 (6.5, 27) | | | 14 (6.75, 34.75) | 15 (9, 29) | 12 (7, 21) | | 15  (8, 29) | | 15.5 (7, 28) | |
| **Number of included studies, n (% total)** | | | | | | | | | | | | | | | | |
| 0 | 3 (0.3) | 0 (0.0) | 0 (0.0) | 0 (0.0) | | 0 (0.0) | | | 2 (4.3) | 0 (0.0) | 0 (0.0) | | 1 (0.7) | | 0 (0.0) | |
| 1-5 | 121 (11.4) | 13 (9.3) | 11 (6.7) | 17 (9.5) | | 6 (14.3) | | | 8 (17.0) | 22 (10.8) | 23 (18.7) | | 17 (12.7) | | 4 (12.9) | |
| 6-15 | 401 (37.8) | 43 (30.7) | 65 (39.9) | 65 (36.3) | | 19 (45.2) | | | 14 (29.8) | 77 (37.9) | 56 (45.5) | | 51 (38.1) | | 11 (35.5) | |
| 16-30 | 244 (23.0) | 38 (27.1) | 37 (22.7) | 42 (23.5) | | 8 (19.0) | | | 9 (19.1) | 50 (24.6) | 22 (17.9) | | 30 (22.4) | | 8 (25.8) | |
| >30 | 241 (22.7) | 36 (25.7) | 45 (27.6) | 40 (22.3) | | 8 (19.0) | | | 13 (27.7) | 45 (22.2) | 18 (14.6) | | 29 (21.6) | | 7 (22.6) | |
| Unclear/Not reported | 52 (4.9) | 10 (7.1) | 5 (3.1) | 15 (8.4) | | 1 (2.4) | | | 1 (2.1) | 9 (4.4) | 4 (3.3) | | 6 (4.5) | | 1 (3.2) | |
| **Number of included RCTs, median (IQR)** | | | | | | | | | | | | | | | | |
|  | 1 (0, 6) | 0 (0, 3.75) | 4 (0, 9) | 1 (0, 6) | | 0 (0, 5) | | | 3 (0, 6) | 3 (0, 9.75) | 0 (0, 3) | | 1 (0, 6) | | 0 (0, 0) | |
| **Number of included RCTs, n (% total)** | | | | | | | | | | | | | | | | |
| 0 | 437 (41.1) | 83 (59.3) | 52 (31.9) | 57 (31.8) | | 21 (50.0) | | | 14 (29.8) | 74 (36.5) | 63 (51.2) | | 48 (35.8) | | 25 (80.6) | |
| 1-2 | 89 (8.4) | 9 (6.4) | 9 (5.5) | 19 (10.6) | | 3 (7.1) | | | 6 (12.8) | 8 (3.9) | 14 (11.4) | | 19 (14.2) | | 2 (6.5) | |
| 3-4 | 61 (5.7) | 7 (5.0) | 9 (5.5) | 7 (3.9) | | 2 (4.8) | | | 6 (12.8) | 17 (8.4) | 5 (4.1) | | 8 (6.0) | | 0 (0.0) | |
| 5-10 | 156 (14.7) | 10 (7.1) | 32 (19.6) | 30 (16.8) | | 6 (14.3) | | | 13 (27.7) | 36 (17.7) | 12 (9.8) | | 17 (12.7) | | 0 (0.0) | |
| 11-20 | 78 (7.3) | 12 (8.6) | 20 (12.3) | 9 (5.0) | | 3 (7.1) | | | 2 (4.3) | 11 (5.4) | 9 (7.3) | | 11 (8.2) | | 1 (3.2) | |
| >20 | 56 (5.3) | 7 (5.0) | 10 (6.1) | 4 (2.2) | | 2 (4.8) | | | 0 (0.0) | 26 (12.8) | 0 (0.0) | | 7 (5.2) | | 0 (0.0) | |
| Unclear/Not reported | 185 (17.4) | 12 (8.6) | 31 (19.0) | 53 (29.6) | | 5 (11.9) | | | 6 (12.8) | 31 (15.3) | 20 (16.3) | | 24 (17.9) | | 3 (9.7) | |
| NCRs, non-Cochrane Reviews; CRs, Cochrane Reviews; IQR, interquartile range, N/A, not applicable. | | | | | | | | | | | | | | | | |

| ***Table S7.*** ***Number of Studies Contributed Data to the Largest Meta-Analysis in Oral Health Systematic Reviews*** | | | | | | | | | | | | | | | | | | | | | | | | | |
| --- | --- | --- | --- | --- | --- | --- | --- | --- | --- | --- | --- | --- | --- | --- | --- | --- | --- | --- | --- | --- | --- | --- | --- | --- | --- |
|  | **Overall**  (No. Overall=1188: NCRs=1062 & CRs=126) | | | **Oral Medicine**  **& Oral Pathology**  (No. Overall=162: NCRs=140 & CRs=22) | | | **Dental Public Health**  (No. Overall=184: NCRs=163 & CRs=21) | | | **Prosthodontics & Restorative Dentistry**  (No. Overall=198: NCRs=179 & CRs=19) | | | **Pediatric Dentistry**  (No. Overall=50: NCRs=42 & CRs=8) | | **Endodontics**  (No. Overall=54: NCRs=47 & CRs=7) | **Periodontics** (No. Overall=212: NCRs=203 & CRs=9) | **Orthodontics &Dentofacial Orthopedics**  (No. Overall=138: NCRs=123 & CRs=15) | | | **Oral and Maxillofacial**  **Surgery** (No. Overall=159: NCRs=134 & CRs=25) | | | **Oral and Maxillo-facial Radiology** (No.Overall=31: NCRs=31 & CRs=0) | | |
| Meta-Analysis Conducted, N (% Total) | | | | | | | | | | | | | | | | | | | | | | | | | |
| ***Overall (Cochrane & Non-Cochrane Reviews)*** | | | | | | | | | | | | | | | | | | | | | | | | | |
| Yes | 518 (43.6) | | | 71 (43.8) | | | 82 (44.6) | | | 82 (41.4) | 16 (32.0) | | | | 31 (57.4) | 120 (56.6) | 43 (31.2) | | | 62 (39.0) | | | 11(35.5) | | |
| No | 670 (56.4) | | | 91 (56.2) | | | 102 (55.4) | | | 116(58.6) | 34 (68.0) | | | | 23 (42.6) | 92 (43.4) | 95 (68.8) | | | 97 (61.0) | | | 20(64.5) | | |
| ***Cochrane Reviews*** | | | | | | | | | | | | | | | | | | | | | | | | | |
| Yes | 64 (50.8) | | | 14 (63.6) | | | 16 (76.2) | | | 6 (31.6) | 3 (37.5) | | | | 2 (28.6) | 7 (77.8) | 4 (26.7) | | | 12 (48.0) | | | 0 (0.0) | | |
| No | 62 (49.2) | | | 8 (36.4) | | | 5 (23.8) | | | 13 (68.4) | 5 (62.5) | | | | 5 (71.4) | 2 (22.2) | 11 (73.3) | | | 13 (52.0) | | | 0 (0.0) | | |
| ***Non-Cochrane Reviews*** | | | | | | | | | | | | | | | | | | | | | | | | | |
| Yes | 454 (42.7) | | | 57 (40.7) | | | 66 (40.5) | | | 76 (42.5) | 13 (31.0) | | | | 29 (61.7) | 113 (55.7) | 39 (31.7) | | | 50 (37.3) | | | 11(35.5) | | |
| No | 608 (57.3) | | | 83 (59.3) | | | 97 (59.5) | | | 103 (57.5) | 29 (69.0) | | | | 18 (38.3) | 90 (44.3) | 84 (68.3) | | | 84 (62.7) | | | 20(64.5) | | |
| Number of Studies Contributed Data to the Largest Meta-Analysis Conducted | | | | | | | | | | | | | | | | | | | | | | | | | |
| ***Overall (Cochrane & Non-Cochrane Reviews)*** | | | | | | | | | | | | | | | | | | | | | | | | | |
| *Total Number* | N=518 | | N=71 | | | N=82 | | | | N=82 | | | N=16 | | N=31 | N=120 | | | N=43 | | | N=62 | | | N=11 |
| **Number of studies in largest meta-analysis, median (IQR)** | | | | | | | | | | | | | | | | | | | | | | | | | |
|  | 9 (5, 18) | | 12 (7, 20.25) | | | 9 (6, 20) | | | | 10 (6, 17.25) | | | 9.5 (4.25, 17.5) | | 8 (5, 13) | 7 (5, 13) | | | 7 (3, 13) | | | 10 (5, 19) | | | 15.5 (4.25,34.5) |
| **Number of studies in largest meta-analysis, n (% total)** | | | | | | | | | | | | | | | | | | | | | | | | | |
| 2-4 | 100 (19.3) | | 11 (15.5) | | | 12 (14.6) | | | | 15 (18.3) | | | 4 (25.0) | | 7 (22.6) | 27 (22.5) | | | 11 (25.6) | | | 11 (17.7) | | | 2 (18.2) |
| 5-10 | 200 (38.6) | | 21 (29.6) | | | 34 (41.5) | | | | 28 (34.1) | | | 5 (31.2) | | 15 (48.4) | 57 (47.5) | | | 16 (37.2) | | | 21 (33.9) | | | 3 (27.3) |
| 11-20 | 108 (20.8) | | 21 (29.6) | | | 15 (18.3) | | | | 24 (29.3) | | | 4 (25.0) | | 3 (9.7) | 15 (12.5) | | | 9 (20.9) | | | 17 (27.4) | | | 0 (0.0) |
| >20 | 104 (20.1) | | 17 (23.9) | | | 20 (24.4) | | | | 15 (18.3) | | | 3 (18.8) | | 6 (19.4) | 19 (15.8) | | | 6 (14.0) | | | 13 (21.0) | | | 5 (45.5) |
| Unclear/Not reported | 6 (1.2) | | 1 (1.4) | | | 1 (1.2) | | | | 0 (0.0) | | | 0 (0.0) | | 0 (0.0) | 2 (1.7) | | | 1 (2.3) | | | 0 (0.0) | | | 1 (9.1) |
| *Total Number* | N=518 | | N=71 | | | N=82 | | | | N=82 | | | N=16 | | N=31 | N=120 | | | N=43 | | | N=62 | | | N=11 |
| **Number of RCTs in largest meta-analysis, median (IQR)** | | | | | | | | | | | | | | | | | | | | | | | | | |
|  | 2 (0, 6) | | 0 (0, 3) | | | 5 (0, 9) | | | 1 (0, 4.25) | | | 2 (0, 5.75) | | 2.5 (0, 6) | | 3 (0, 7) | | 1 (0, 3.25) | | | 3.5 (0, 8) | | | 0 (0, 0) | |
| **Number of RCTs in largest meta-analysis, n (% total)** | | | | | | | | | | | | | | | | | | | | | | | | | |
| 0 | 188 (36.3) | 45 (63.4) | | | 18 (22.0) | | | 29 (35.4) | | | | 6 (37.5) | | 8 (25.8) | | 39 (32.5) | | 16 (37.2) | | | 17 (27.4) | | | 10(90.9) | |
| 2-4 | 107 (20.7) | 11 (15.5) | | | 15 (18.3) | | | 18 (22.0) | | | | 5 (31.2) | | 10 (32.3) | | 23 (19.2) | | 11 (25.6) | | | 14 (22.6) | | | 0 (0.0) | |
| 5-10 | 104 (20.1) | 10 (14.1) | | | 23 (28.0) | | | 12 (14.6) | | | | 0 (0.0) | | 6 (19.4) | | 33 (27.5) | | 7 (16.3) | | | 12 (19.4) | | | 1 (9.1) | |
| 11-20 | 27 (5.2) | 1 (1.4) | | | 7 (8.5) | | | 3 (3.7) | | | | 3 (18.8) | | 0 (0.0) | | 5 (4.2) | | 0 (0.0) | | | 8 (12.9) | | | 0 (0.0) | |
| >20 | 21 (4.1) | 1 (1.4) | | | 8 (9.8) | | | 0 (0.0) | | | | 0 (0.0) | | 2 (6.5) | | 9 (7.5) | | 0 (0.0) | | | 1 (1.6) | | | 0 (0.0) | |
| Unclear/Not reported | 71 (13.7) | 3 (4.2) | | | 11 (13.4) | | | 20 (24.4) | | | | 2 (12.5) | | 5 (16.1) | | 11 (9.2) | | 9 (20.9) | | | 10 (16.1) | | | 0 (0.0) | |
| ***Cochrane Reviews*** | | | | | | | | | | | | | | | | | | | | | | | | | |
| *Total Number* | N=64 | N=14 | | | N=16 | | | N=6 | | | | N=3 | | N=2 | | N=7 | | N=4 | | | N=12 | | | N=0 | |
| **Number of studies in largest meta-analysis, median (IQR)** | | | | | | | | | | | | | | | | | | | | | | | | | |
|  | 5.5 (3, 9) | 6.5 (3, 9) | | | 9 (6.25, 31.25) | | | 3 (2, 4.25) | | | | 2 (2, 11) | | 5 (2, 8) | | 3 (2, 9) | | 2.5 (2, 3) | | | 4 (2.25, 13) | | | N/A | |
| **Number of studies in largest meta-analysis, n (% total)** | | | | | | | | | | | | | | | | | | | | | | | | | |
| 2-4 | 31 (48.4) | 6 (42.9) | | | 2 (12.5) | | | 5 (83.3) | | | | 2 (66.7) | | 1 (50.0) | | 4 (57.1) | | 4 (100) | | | 7 (58.3) | | | 0 (0.0) | |
| 5-10 | 20 (31.2) | 8 (57.1) | | | 7 (43.8) | | | 1 (16.7) | | | | 0 (0.0) | | 1 (50.0) | | 2 (28.6) | | 0 (0.0) | | | 1 (8.3) | | | 0 (0.0) | |
| 11-20 | 7 (10.9) | 0 (0.0) | | | 2 (12.5) | | | 0 (0.0) | | | | 1 (33.3) | | 0 (0.0) | | 1 (14.3) | | 0 (0.0) | | | 3 (25.0) | | | 0 (0.0) | |
| >20 | 6 (9.4) | 0 (0.0) | | | 5 (31.2) | | | 0 (0.0) | | | | 0 (0.0) | | 0 (0.0) | | 0 (0.0) | | 0 (0.0) | | | 1 (8.3) | | | 0 (0.0) | |
| Unclear/Not reported | 0 (0.0) | 0 (0.0) | | | 0 (0.0) | | | 0 (0.0) | | | | 0 (0.0) | | 0 (0.0) | | 0 (0.0) | | 0 (0.0) | | | 0 (0.0) | | | 0 (0.0) | |
| *Total Number* | N=64 | N=14 | | | N=16 | | | N=6 | | | | N=3 | | N=2 | | N=7 | | N=4 | | | N=12 | | | N=0 | |
| **Number of RCTs in largest meta-analysis, median (IQR)** | | | | | | | | | | | | | | | | | | | | | | | | | |
|  | 4.5 (2, 9) | 6.5 (3, 9) | | | 8 (6, 31.25) | | | 2.5 (2, 4.25) | | | | 2 (2, 11) | | 5 (2, 8) | | 3 (2, 9) | | 2.5 (2, 3) | | | 4 (2.25, 13) | | | N/A | |
| **Number of RCTs in largest meta-analysis, n (% total)** | | | | | | | | | | | | | | | | | | | | | | | | | |
| 0 | 0 (0.0) | 0 (0.0) | | | 0 (0.0) | | | 0 (0.0) | | | | 0 (0.0) | | 0 (0.0) | | 0 (0.0) | | 0 (0.0) | | | 0 (0.0) | | | 0 (0.0) | |
| 2-4 | 32 (50.0) | 6 (42.9) | | | 3 (18.8) | | | 5 (83.3) | | | | 2 (66.7) | | 1 (50.0) | | 4 (57.1) | | 4 (100) | | | 7 (58.3) | | | 0 (0.0) | |
| 5-10 | 19 (29.7) | 8 (57.1) | | | 6 (37.5) | | | 1 (16.7) | | | | 0 (0.0) | | 1 (50.0) | | 2 (28.6) | | 0 (0.0) | | | 1 (8.3) | | | 0 (0.0) | |
| 11-20 | 7 (10.9) | 0 (0.0) | | | 2 (12.5) | | | 0 (0.0) | | | | 1 (33.3) | | 0 (0.0) | | 1 (14.3) | | 0 (0.0) | | | 3 (25.0) | | | 0 (0.0) | |
| >20 | 6 (9.4) | 0 (0.0) | | | 5 (31.2) | | | 0 (0.0) | | | | 0 (0.0) | | 0 (0.0) | | 0 (0.0) | | 0 (0.0) | | | 1 (8.3) | | | 0 (0.0) | |
| Unclear/Not reported | 0 (0.0) | 0 (0.0) | | | 0 (0.0) | | | 0 (0.0) | | | | 0 (0.0) | | 0 (0.0) | | 0 (0.0) | | 0 (0.0) | | | 0 (0.0) | | | 0 (0.0) | |
| ***Non-Cochrane Reviews*** | | | | | | | | | | | | | | | | | | | | | | | | | |
| *Total Number* | N=454 | N=57 | | | N=66 | | | N=76 | | | | N=13 | | N=29 | | N=113 | | N=39 | | | N=50 | | | N=11 | |
| **Number of studies in largest meta-analysis, median (IQR)** | | | | | | | | | | | | | | | | | | | | | | | | | |
|  | 9 (6, 19) | 15 (9, 22.75) | | | 9 (6, 19) | | | 11 (7, 18) | | | | 10 (5.5, 20) | | 8 (5, 16) | | 7 (5, 13) | | 8 (5, 13) | | | 11.5 (6.75, 20.5) | | | 15.5 (4.25, 34.5) | |
| **Number of studies in largest meta-analysis, n (% total)** | | | | | | | | | | | | | | | | | | | | | | | | | |
| 2-4 | 69 (15.2) | 5 (8.8) | | | 10 (15.2) | | | 10 (13.2) | | | | 2 (15.4) | | 6 (20.7) | | 23 (20.4) | | 7 (17.9) | | | 4 (8.0) | | | 2 (18.2) | |
| 5-10 | 180 (39.6) | 13 (22.8) | | | 27 (40.9) | | | 27 (35.5) | | | | 5 (38.5) | | 14 (48.3) | | 55 (48.7) | | 16 (41.0) | | | 20 (40.0) | | | 3 (27.3) | |
| 11-20 | 101 (22.2) | 21 (36.8) | | | 13 (19.7) | | | 24 (31.6) | | | | 3 (23.1) | | 3 (10.3) | | 14 (12.4) | | 9 (23.1) | | | 14 (28.0) | | | 0 (0.0) | |
| >20 | 98 (21.6) | 17 (29.8) | | | 15 (22.7) | | | 15 (19.7) | | | | 3 (23.1) | | 6 (20.7) | | 19 (16.8) | | 6 (15.4) | | | 12 (24.0) | | | 5 (45.5) | |
| Unclear/Not reported | 6 (1.3) | 1 (1.8) | | | 1 (1.5) | | | 0 (0.0) | | | | 0 (0.0) | | 0 (0.0) | | 2 (1.8) | | 1 (2.6) | | | 0 (0.0) | | | 1 (9.1) | |
| *Total Number* | N=454 | N=57 | | | N=66 | | | N=76 | | | | N=13 | | N=29 | | N=113 | | N=39 | | | N=50 | | | N=11 | |
| **Number of RCTs in largest meta-analysis, median (IQR)** | | | | | | | | | | | | | | | | | | | | | | | | | |
|  | 1 (0, 6) | 0 (0, 0) | | | 4 (0, 8) | | | 0 (0, 4.75) | | | | 0 (0, 4) | | 2.5 (0, 5.75) | | 3 (0, 7) | | 0 (0, 2.25) | | | 2.5 (0, 7) | | | 0 (0, 0) | |
| **Number of RCTs in largest meta-analysis, n (% total)** | | | | | | | | | | | | | | | | | | | | | | | | | |
| 0 | 188 (41.4) | 45 (78.9) | | | 18 (27.3) | | | 29 (38.2) | | | | 6 (46.2) | | 8 (27.6) | | 39 (34.5) | | 16 (41.0) | | | 17 (34.0) | | | 10(90.9) | |
| 2-4 | 75 (16.5) | 5 (8.8) | | | 12 (18.2) | | | 13 (17.1) | | | | 3 (23.1) | | 9 (31.0) | | 19 (16.8) | | 7 (17.9) | | | 7 (14.0) | | | 0 (0.0) | |
| 5-10 | 85 (18.7) | 2 (3.5) | | | 17 (25.8) | | | 11 (14.5) | | | | 0 (0.0) | | 5 (17.2) | | 31 (27.4) | | 7 (17.9) | | | 11 (22.0) | | | 1 (9.1) | |
| 11-20 | 20 (4.4) | 1 (1.8) | | | 5 (7.6) | | | 3 (3.9) | | | | 2 (15.4) | | 0 (0.0) | | 4 (3.5) | | 0 (0.0) | | | 5 (10.0) | | | 0 (0.0) | |
| >20 | 15 (3.3) | 1 (1.8) | | | 3 (4.5) | | | 0 (0.0) | | | | 0 (0.0) | | 2 (6.9) | | 9 (8.0) | | 0 (0.0) | | | 0 (0.0) | | | 0 (0.0) | |
| Unclear/Not reported | 71 (15.6) | 3 (5.3) | | | 11 (16.7) | | | 20 (26.3) | | | | 2 (15.4) | | 5 (17.2) | | 11 (9.7) | | 9 (23.1) | | | 10 (20.0) | | | 0 (0.0) | |
| NCRs, non-Cochrane Reviews; CRs, Cochrane Reviews; IQR, interquartile range, N/A, not applicable. | | | | | | | | | | | | | | | | | | | | | | | | | |
